# Cross-Linked Poly(Ethylene Carbonate/Ethylene Oxide) Electrolyte for Solid-State Li-Ion Batteries Exploiting Membrane-Electrode Assembly

**DOI:** 10.1021/acspolymersau.6c00006

**Published:** 2026-06-03

**Authors:** Nantapat Soontornnon, Edoardo Barcaro, Shuto Takayama, Kento Kimura, Yoichi Tominaga, Jusef Hassoun

**Affiliations:** † Institute of Global Innovation Research (GIR), 13125 Tokyo University of Agriculture and Technology, 3-8-1 Harumi-cho, Fuchu-shi, Tokyo 183-8538, Japan; ‡ Department of Applied Chemistry, Graduate School of Engineering, Tokyo University of Agriculture and Technology, 2-24-16 Naka-cho, Koganei-shi, Tokyo 184-8588, Japan; § Graduate School of Advanced Interdisciplinary Science, Tokyo University of Agriculture and Technology, 2-24-16, Nakamachi, Koganei-shi, Tokyo 184-8588, Japan; ∥ Department of Chemical, Pharmaceutical and Agricultural Sciences University of Ferrara, Via Fossato di Mortara 17, Ferrara 44121, Italy; ⊥ Center for Nanotechnology Innovation @NEST, Istituto Italiano di Tecnologia, Piazza San Silvestro 12, Pisa I-56127, Italy

**Keywords:** PEC, PEO, cross-linked polymer electrolyte, MEA, Li-ion battery

## Abstract

Safety concerns of flammable liquid electrolytes as well as challenges related to the use of high-capacity anodes, such as silicon-based ones, are among the bottlenecks for achieving next-generation Li-ion batteries. Herein, we synergistically blend poly­(ethylene carbonate) and poly­(ethylene oxide) copolymers with complementary molecular weights and cross-linking densities into membranes with lithium bis­(fluorosulfonyl)­imide (LiFSI) salt concentrated up to 140 mol %. The process leads to membranes without any electrochemical performance-enhancing additive, denoted as additive-free in this work. The thin, mechanically robust, and self-standing cross-linked polymer electrolyte membrane (CP-EM) isused in an all-solid-state battery (ASSB), with scalable LiFePO_4_ membrane-electrode assembly (LFP-MEA), and a prelithiated silicon oxide-carbon (Li_*y*_SiO_*x*_C) anode. Electrochemical characterization reveals a CP-EM with a high lithium-ion transference number (*t* ^+^) of 0.61, a low activation energy (*E*_a_) of 0.110 eV, anodic stability exceeding 4.0 V, and a suitable interphase with the lithium metal. The integration of the CP-EM into the proof-of-concept full cell with the LFP-MEA and Li_*y*_SiO_*x*_ is conducted with a practical N/P ratio of 1.16. The full cell delivers an initial reversible capacity of ∼140 mAh g^–1^ at 70 °C, with an average working voltage of 3.2 V and limited polarization. Despite stability and rate capability still needing further improvements, this study offers a synergic design for blending distinct macromolecular architectures, with a specific balance between segmental flexibility for ion transport and structural integrity. The outcomes demonstrate the practical viability of the integrated MEA in scalable full cells operating at relatively high temperatures.

## Graphical abstract

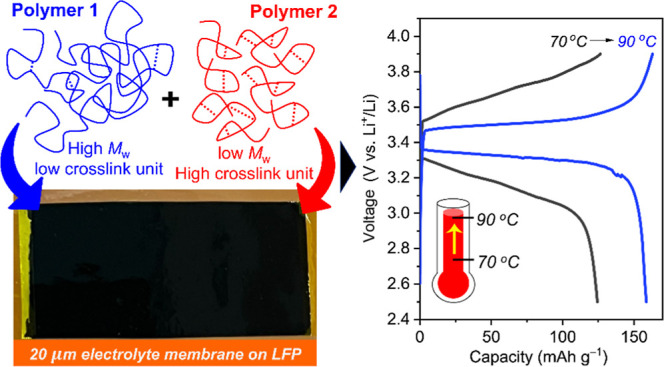

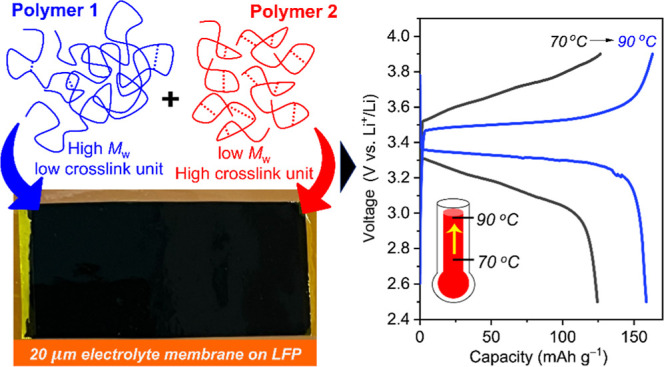

## 1 Introduction

Presently, most commercial batteries are based on Li-ion chemistry (LIBs) employing liquid electrolytes,^1,2^ a breakthrough that was recognized with the 2019 Nobel Prize in Chemistry. Driven by the increasing demand for lightweight and high-energy-density electronic devices, battery safety has become a paramount concern for both consumers and manufacturers, due to possible leakage of liquid electrolytes and thermal runaway issues.^3,4^ Therefore, solid-state lithium-ion batteries (SSLIBs) have emerged as a key technology, offering enhanced thermal stability and mitigating the risks associated with flammable liquid electrolytes. Among solid-state electrolytes, solid polymer ones (SPEs) are highly attractive for next-generation wearable energy storage systems since they dissolve lithium salts in polar polymers with intrinsic flexibility and processability.^5^ The concept of polymer-based ion conduction was initially demonstrated through poly­(ethylene oxide) (PEO)/metal salt complexes since the low glass-transition temperature (*T*_g_) of the above polymer imparts fast Li^+^ transport mainly occurring in the amorphous phase, with an ionic conductivity (σ) of 10^–4^ S cm^–1^ and *t* ^+^ values from 0.2 to 0.4 at working temperatures higher than 60 °C in practical applications.^6,7^ Although the ionic conductivity value of PEO-SPE is lower than that of commercial liquid electrolytes (σ > 10^–3^ S cm^–1^), various studies proposed this system as a typical solid polymer electrolyte, and investigations into PEO-based SPEs have predominantly focused on Li-metal batteries (LMBs).^8,9^ However, the practical use of Li metal was severely hindered by safety risks associated with dendrite growth and possible short circuits.^10^ In this view, the transition from Li metal-based SSLIBs to systems using alternative anodes, such as silicon (Si) with a theoretical capacity as high as 4200 mAh g^–1^, appeared to be a strategic compromise to improve the battery safety.^11,12^ However, integrating these alloy anodes with SPEs appeared quite challenging due to substantial volume changes and interfacial instability.^13^ Despite a recent study successfully demonstrating a PEO-SPE in a Si/C anode half-cell, this system required the incorporation of an ionic liquid to ensure adequate compatibility and electrochemical performance.^14^ Furthermore, there have been various reports demonstrating the practical application of poly­(ether)-based electrolytes in Li-ion full-cell systems incorporating inorganic ceramic fillers, additives, and­(or) throughout complex assembly processes intended to increase the electrochemical properties of SPE.^15–19^ Recently, state-of-the-art systems have improved PEO-based SPE to achieve a conductivity of ∼10^–4^ S cm^–1^ at a temperature near 60 °C, with *t* ^+^ values higher than 0.6.^20^ On the other hand, additive-free all-solid-state full cells are currently limited, particularly in systems using host polymers other than the typical polyethers.

CO_2_-derived aliphatic polycarbonates, such as poly­(ethylene carbonate) (PEC), exhibited enhanced ionic conductivity and electrochemical stability with increasing salt concentration as demonstrated by our previous reports.^21–25^ Besides, this PEC-SPE exhibited a higher *t* ^+^ value of ∼0.5 compared to a typical PEO-SPE.^21^ Building on this, we have developed poly­(ethylene carbonate/ethylene oxide) (P­(EC/EO)) copolymer electrolytes that showed a superior ionic conductivity and *t* ^+^ of 4.8 × 10^–4^ S cm^–1^ and 0.66, respectively, compared to their PEC and PEO counterparts.^26,27^ The higher polarity and dielectric constant of carbonate groups in PEC compared to ether groups in PEO can enhance the salt dissociation and increase the concentration of free charge carriers. On the other hand, PEO may provide strong coordination sites for Li^+^ through ether oxygens, facilitating ion mobility along polymer chains, but can often suffer from limited salt dissociation and restricted segmental motion, especially at low temperatures due to its semicrystalline nature. Furthermore, the random sequence of the PEC/PEO copolymer can decrease the crystallinity of PEO and improve segmental mobility, further promoting ionic conductivity and Li^+^ transport. Hence, by combining the advantages of PEC and PEO, the system may benefit from both enhanced salt dissociation as well as efficient Li^+^ coordination and transport pathways.^27^ However, these polymers suffered from poor mechanical integrity and excessive tackiness in high-salt regimes, which have been successfully addressed by cross-linking with allyl glycidyl ether (AGE) to get a robust poly­(ethylene carbonate-*co*-ethylene oxide-*co*-allyl glycidyl ether carbonate-*co*-allyl glycidyl ether oxide (P­(EC/EO/AGEC/AGEO)) electrolyte.^28^ While we previously achieved a self-standing membrane capable of stable cycling at 40 °C in a half-cell with LFP operating at C/10, the substantial membrane thickness (∼400 μm) resulted in high polarization, posing a significant technical challenge.^28^ Subsequently, to address interfacial compatibility, we explored an in situ cross-linking strategy wherein cross-linked polymer precursor functioned as a cathode binder.^29^ Although this approach improved the electrode/electrolyte contact, the resulting electrolyte layer remained relatively thick (∼300 μm) to achieve a mechanically stable electrolyte membrane. Therefore, the optimization of the thickness required for practically handling the cell and achieving the Li-ion version of the battery represented a significant challenge, especially with a silicon-based anode. We adopted in this work the MEA strategy, previously established for high-molecular-weight composite PEO (*M*_w_ = 600 k Da) electrolyte,^30^ to achieve a stable composite electrolyte membrane directly on the cathode tape. While this method was proven to enhance interfacial contact in PEO-based composite polymers, its implementation here represented a novel pathway to get an additive-free cross-linked electrolyte membrane under high-salt concentration. With this respect, we synergistically blended two distinct copolymers with complementary molecular weights and cross-linking unit densities. This formulation was specifically designed to optimize the solution viscosity of the electrolyte precursor, facilitating the direct casting of a thin, mechanically robust electrolyte layer. Comprehensive chemical, thermal, and electrochemical characterizations have been utilized to confirm the stability and transport properties of the resulting membrane. Furthermore, the practical viability of the CP-EM has been verified through a preliminary evaluation, ranging from Li-metal half-cells to the most challenging all-solid-state full-cell, pairing high-capacity Li_*y*_SiO_*x*_C anodes^31^ with LFP-MEA.^32^

## 2 Experimental Section

### 2.1 Chemicals

Ethylene oxide (EO, >99.9%, Air Water Inc.), carbon dioxide (CO_2_, 99.99%, Nippon Sanso), allyl glycidyl ether (AGE, TCI Co.), methanol (CH_3_OH, >99.8%, Kanto Chemical Co.), hexane (C_6_H_14_, >96%, Kanto Chemical Co.), dichloromethane (CH_2_Cl_2_, >99.5%, Kanto Chemical Co.), dehydrated acetonitrile (ACN, CH_3_CN, Sigma-Aldrich), deuterated chloroform (CDCl_3_, 99.8 atom % D with 0.03 vol % tetramethylsilane (TMS)), polyvinylidene fluoride (PVdF, Solef 6020), LFP (carbon content 5%, Advanced Lithium Electrochemistry, Aleees Taiwan, model A1100),^32^ 1 M lithium hexafluoro phosphate (LiPF_6_) in ethylene carbonate/dimethyl carbonate (EC/DMC) 50:50% V/V (LP30, battery grade, Sigma-Aldrich), Super P carbon (SPC, Timcal), *N*-methyl-2-pyrrolidone (NMP, Sigma-Aldrich), and diphenyl­(2,4,6-trimethylbenzoyl)­phosphine oxide (TPO, TCI Co.) were used as received. LiFSI (battery-grade, Kishida Chemical Co.) was further dried in a Büchi oven at 110 °C for 48 h to remove possible water and then transferred to an argon (Ar)-filled glovebox (MBraun, O_2_ and H_2_O contents <1 ppm) before use. The silicon oxide-carbon powder (SiO_*x*_C) was used as prepared according to our previous work.^31^

### 2.2 Synthesis of P­(EC/EO/AGEC/AGEO) Copolymers

The detailed copolymerization procedure is also described in our previous work.^28^ In brief, two types of P­(EC/EO/AGEC/AGEO) copolymers were synthesized via copolymerization of CO_2_, EO, and AGE. The EO/AGE molar ratios were set to 10:1 and 2:1, respectively, in a total volume of 5 mL, while CO_2_ was introduced with an overall pressure of 4 MPa in the system. The reactions were catalyzed by a double metal cyanide catalyst (0.001 g per mL of total monomers, EO + AGE) at 60 °C for 20 h with a stirring speed of 600 rpm. The resulting copolymers were dissolved in chloroform (CHCl_3_) containing 2–3 drops of 0.05 M HCl­(aq) to terminate the reaction. The obtained products were purified by precipitation, achieved by slowly adding the polymer solution to an excess mixture of methanol and hexane (MeOH/hexane). The whole preparation process was repeated three times. The purified copolymer was then dissolved in dichloromethane (DCM) and filtered through a 1 μm glass microfilter using a 10 mL syringe, before being cast onto a PTFE Petri dish. The cast solutions were dried under vacuum at 40 °C for 48 h to obtain polymer films (neat copolymers).

### 2.3 Chemical Characterization of P­(EC/EO/AGEC/AGEO) Copolymers

The chemical structure of the neat P­(EC/EO/AGEC/AGEO) was detected by ^1^H NMR measurement using a JNM-ECX400 spectrometer (JEOL Ltd.) at a sample concentration of 1 w/v %. The neat copolymer was dissolved in CDCl_3_. The molecular weight and polydispersity index (PDI) of the purified copolymer were analyzed via size exclusion chromatography (SEC). The samples were prepared by dissolving the copolymer in chloroform (1 mg of copolymer/1 mL of chloroform) and then examined using an HLC-8320 GPC EcoSEC (TOSOH Co.). Reported molecular masses of copolymers were calibrated with narrow molar mass polystyrene standards (PDI: 1.0–1.2).

### 2.4 Preparation of the CP-EM

The above dried synthetic copolymers, i.e., the neat P­(EC/EO/AGEC/AGEO), were used as precursors to prepare the CP-EM using a solution casting method. Two P­(EC/EO/AGEC/AGEO) polymers with molecular weights of *M*_*n*_ = 140 k (neat_140k_) and 28 k (neat_28k_), with a mass ratio of 50:50, were dissolved in acetonitrile (ACN) under ambient conditions in a glass vial with a magnetic stirrer. The mixture was stirred at room temperature until complete dissolution of the polymers. Subsequently, LiFSI was weighed inside an Ar-filled glovebox (MBraun, O_2_ and H_2_O contents <1 ppm) to avoid mass errors caused by moisture uptake. The weighed LiFSI was then transferred outside the glovebox and added to the polymer solution with 140 mol %, corresponding to (moles of LiFSI)/(total moles of EC + EO + AGEC + AGEO repeating units) × 100, for each polymer. A salt concentration of 140 mol % was selected as a suitable balance between efficient ion conduction and mechanical integrity, as demonstrated in our previous study, which exhibited an ionic conductivity higher than 10^–5^ S cm^–1^ at 70 °C, while holding sufficient mechanical properties of the membrane structure at room temperature.^28^ Any residual LiFSI in the vial was rinsed with a small amount of ACN and poured into the polymer solution to ensure complete transfer. The total solvent amount was 0.45 mL of ACN per 0.1 g of polymer. The polymer/salt mixture was further stirred at room temperature for 1 h to obtain the polymer/salt homogeneous solution. Afterward, the photoinitiator TPO (cross-linking agent) was added to the polymer/salt solution at 3 wt % with respect to the polymer content. The resulting mixture was stirred for 10 min to obtain a homogeneous and viscous solution, which was the electrolyte precursor. The CP-EM was then achieved by casting the electrolyte precursor onto a fluorinated ethylene propylene (FEP) substrate (235FEP-2–50, CS Hyde) using a doctor blade tool (MTI Corp.) set with a gap adjustment of 600 μm. The cast film was subsequently exposed to UV irradiation (365 nm, 50 Hz, 8 W; As one Co.) for 30 min to induce cross-linking reaction. Subsequently, the gel-like cross-linked membrane on the FEP substrate was dried at 70 °C in a vacuum oven for 1 h, and further in a Büchi oven under vacuum at 60 °C for 24 h. The photographs of all preparation steps are depicted in Figure S1 (Supporting Information). The dried membrane was then transferred into an argon (Ar)-filled glovebox (MBraun, O_2_ and H_2_O contents <1 ppm), heated on a plate at 70 °C for 1 h, and then stored for at least 24 h to allow the polymer film to equilibrate by Ar gas absorption. The average thickness of the dry membrane was estimated to be ∼70 μm by gauge.

### 2.5 Electrode Film Preparation

The LFP cathode, the SiO_*x*_C anode, and carbon-based electrodes for specific use were prepared by the slurry-casting method. For the cathode, LFP powder was employed as the active material, PVdF as the binder, and SPC as the conductive agent, with a weight ratio of 80:10:10. All components were weighed in the same beaker, then transferred to a mortar and thoroughly mixed, and subsequently dispersed/dissolved in NMP solvent to obtain a uniform slurry. The homogeneous slurry was cast onto a 15 μm-thick aluminum foil current collector (MTI Corp.) using a doctor blade with a 300 μm gap from the foil surface. The cast cathode was dried in a vacuum oven at 70 °C for 2 h to remove the NMP solvent and water. The dried electrode was then calendared using a rolling machine (MSK-2150, MTI Corp.) to 70% of its initial thickness, yielding a final electrode thickness of approximately 100 μm (including Al current collector). Bare Al was employed to estimate the average mass loading of the LFP cathode, which corresponded to approximately 6 mg cm^–2^. This moderate loading, while lower than that of the commercial batteries, was chosen to ensure reliable electrochemical characterization of the newly developed cross-linked electrolyte without being limited by excessive internal resistance or mechanical failure of the composite electrode during initial testing. Regarding scalability, we envision several strategies to reach practical levels (target area capacity >2.5 mAh cm^–2^ or approximately 15 mg cm^–2^ in terms of cathode mass loading for an all-solid-state battery) close to the commercial Li-ion battery,^33^ such as integrating this CP-EM directly as an electrolyte/binder within the thick cathode architecture to establish a continuous ion-conducting network. Carbon electrodes were prepared following the same procedure as the LFP cathode, with a component weight ratio of SPC/PVdF of 80:20. The SPC-based slurry was cast onto either 15 μm-thick aluminum (SPC-Al) or 30 μm-thick copper foil (SPC-Cu) current collectors (MTI Corp.), depending on the intended electrochemical study (the final thickness of the SPC-based electrodes was measured to be around 70 μm by gauge). The SiO_*x*_C powder was achieved by a hydrothermal synthesis procedure described elsewhere.^31^ The anode film was obtained by casting a slurry onto Cu foil as reported above, with a weight ratio of 80 wt % of active material powder (SiO_*x*_C), 10 wt % PVdF polymer binder, and 10 wt % SPC electron conductors dispersed in NMP solvent. The final thickness of the SiO_*x*_C anode was about 170 μm, while its loading ranged from 3.2 to 3.7 mg cm^–2^.

### 2.6 Thermal, Spectroscopic, and Electrochemical Analysis of the CP-EM

Thermogravimetric analysis (TGA) was performed from 25 to 800 °C using a heating rate of 5 °C min^–1^ under dry N_2_ flow (50 mL min^–1^) with a Mettler-Toledo TGA instrument (Mettler-Toledo, Columbus, OH, USA). The CP-EM sample was prepared in an Ar-filled glovebox by weighing the alumina crucible with cap, then transferred into the TGA tool, limiting the air-exposure time. The reference LiFSI and copolymer without salt were prepared for TGA by using the same procedure as for CP-EM. TGA was also employed to investigate the influence of gas absorption on the CP-EM. The measurement was carried out with isothermal steps at 30 and 70 °C, each maintained for 15 min under dry N_2_ flow (50 mL min^–1^).

Differential scanning calorimetry (DSC) measurement was carried out using a DSC7020 (Hitachi High-Tech Co., Tokyo, Japan) achieving a temperature range from −65 to 145 °C, at a heating/cooling rate of 10 °C min^–1^, and repeated for 3 cycles to remove the thermal history of the CP-EM under a nitrogen (N_2_) flow of 40 mL min^–1^.

Fourier transform infrared (FT-IR) spectroscopy was performed using an FT-IR-4100, Jasco, Japan, equipped with a ZnSe ATR crystal to analyze the chemical structures of the electrolytes. Data were collected under an N_2_ atmosphere at a 4 cm^–1^ resolution for 256 scans. The broad spectral range of 4000–700 cm^–1^ was scanned, while the specific regions between 1800 and 1680 cm^–1^ and 900 and 800 cm^–1^ were deconvoluted to investigate the carbonate and FSI anion peaks, respectively. Peak deconvolution was conducted to evaluate peak areas using Origin Pro software as follows. The FT-IR spectral deconvolution was performed using a Gaussian–Lorentzian function after baseline correction (normalized data, *Y* = 0). To ensure physical validity, strict constraints were applied to the fitting parameters. Specifically, peak centers were restricted within ±2 cm^–1^ of their original positions, and the full-width at half-maximum (FWHM) boundaries were explicitly set by referencing the actual peak width of the reference peak from the pristine polymer. Mechanical strength was assessed using a Sentec OZ502 small tensile tester equipped with a 50 N load cell. The tests were performed on five independent samples of CP-EM. Measurements were carried out at ambient temperature inside an Ar-filled glovebox using a stretching rate of 10 mm min^–1^. The Young modulus for the CP-EM specimen was extracted from the slope of the initial linear stress–strain curve.

A VersaSTAT MC Princeton Applied Research (PAR-AMETEK) instrument was employed to evaluate the ionic conductivity, Li-metal interfacial stability, the lithium transference number (*t* ^+^), and the anodic and cathodic stability of the CP-EM in a 2032 coin-cell (MTI Crop.) configuration. The ionic conductivity was determined using electrochemical impedance spectroscopy (EIS) with parallel 16 mm-diameter stainless-steel disks (SS) as blocking electrodes (SS|CP-EM|SS) and an O-ring (outer diameter = 16 mm, inner diameter = 8 mm) made by poly­(ethylene terephthalate) (PET) sheets (100 μm-thick) to hold the membrane in a CR2032 coin cell. The CP-EM sample was first cut with the stainless-steel handle punch with a diameter size of 8 mm (the membranes were chosen to fit the O-ring dimension) and then placed into the center of the O-ring. The CP-EM cells used for ionic conductivity measurements were heated at 98 °C for 24 h to stabilize the interfacial contact between the electrolyte and SS electrodes. The ionic conductivity was measured during the first cooling from 98 to 30 °C, with 2–3 °C temperature decrements (after each temperature change, the cell was equilibrated for 3 h to ensure thermal stability prior to the EIS measurement) in the frequency range of 50 kHz to 100 Hz with an amplitude of 50 mV. The bulk resistance (*R*_b_) of the CP-EM was determined by fitting the Nyquist plots using Zsimpwin software. Different equivalent circuits were employed depending on the temperature range. An *R*_b1_ *Q*_g_ equivalent circuit was used in the temperature range of 98–72 °C, while an *R*_b1_(*R*_b2_ *Q*_b2_)*Q*_g_ equivalent circuit was used for 70–30 °C. In these models, *R*_b_ (represented by the *R*_b1_ or *R*_b1_ + *R*_b2_ elements) corresponded to the high-frequency region,^34^ *Q*_b_ denoted the constant phase element (CPE) accounting for nonideal capacitive behavior,^35,36^ and *Q*_g_ indicated the cell geometric capacity.^37^ The ionic conductivity was calculated using eq 1 as follows

1 $$\sigma =\frac{l}{R_{b}\times A}$$

where σ represents the ionic conductivity (S cm^–1^), *R*_b_, the bulk resistance obtained from the equivalent circuit fitting, *l*, the thickness of the O-ring spacer (100 μm), and *A*, its area defined by the internal diameter of 8 mm (*A* = 0.5 cm^2^).

The interfacial stability of the Li/CP-EM was also evaluated by EIS using a Li|CP-EM|Li symmetric cell configuration. Li metal foils (∼250 μm thick) were laminated onto the CP-EM membrane surface and aged for at least 24 h prior to use, to ensure contact stabilization between Li and the polymer electrolyte. Then, the Li/CP-EM was punched into discs of 14 mm and 8 mm in diameter. An FEP O-ring (23-5FEP-2-50, thickness = 127 μm, CS Hyde) was placed on the larger (14 mm diameter) Li/CP-EM disc at the polymer side, and the smaller (8 mm-diameter) Li/CP-EM disc was then positioned to fit within the inner diameter of the O-ring, forming a well-aligned Li/polymer/Li contact area. Although the FEP may not be suitable for Li due to possible side reactions of the fluorinated structure, the presence of the membrane prevented its direct contact with the metal and allowed its use. The cells were aged at 70 °C in a Büchi oven, with temperature monitored by a thermocouple (maximum fluctuation ±0.1 °C). The interfacial resistance was initially measured within 12 h after assembly and subsequently monitored daily for 1 week using an EIS frequency range of 500 kHz to 100 mHz and a voltage amplitude of 30 mV. The cell configuration used for determining the *t* ^*+*^ of the CP-EM was identical to that used for interfacial resistance measurements (Li|CP-EM|Li). The *t* ^+^ was determined by the Bruce–Vincent–Evans method^38^ through potentiostatic polarization, with a chronoamperometry performed by applying a DC bias of 30 mV to the symmetric cell. AC impedance spectra were recorded before and after polarization over the frequency range of 500 kHz to 0.1 Hz, with an amplitude of 10 mV at 70 °C. The *t* ^+^ was calculated using eq 2 as follows

2 $$t^{+}=\frac{I_{ss}\times \left(V-I_{0}R_{0}\right)}{I_{0}\times \left(V-I_{ss}R_{ss}\right)}$$

where *I*_0_ is the initial current, *I*_ss_ is the steady-state current, *R*_0_ is the initial resistance of the Li/electrolyte interface before polarization, and *R*_ss_ is the steady-state resistance of the Li/electrolyte interface after the polarization. In this respect, the resistance values of the Li/electrolyte interface before and after polarization were fitted using a nonlinear least-squares (NLLS) method with the aid of Boukamp software.^35,36^ The Nyquist plots for interfacial stability and *t* ^+^ determination were fitted to the *R_b_* (*R*_2_ *Q*_2_)­(*R*_3_ *Q*_3_)­(*R*_4_ *Q*_w_) equivalent circuit. In this circuit, *R*_b_ represented the resistance of the CP-EM as intercept at the high frequency, *R*_2_ + *R*_3_ + *R*_4_ corresponded to the overall resistance of the semicircles at the middle-frequency, accounting for the electrode/electrolyte interphase, film formation, and charge transfer resistance (*R_i_*), *Q*_*i*_ (*i* = 1, 2, 3) represented the related CPE, while *Q*_w_ indicated the Warburg element accounting for the Li^+^ ions diffusion at low frequency.^39^ Only fits with a χ^2^ values of the order of 10^–4^ or lower were considered reliable for subsequent calculations. Lithium plating/stripping tests were conducted at 70 °C in a Li|CP-EM|Li symmetric cell by galvanostatic cycling at a current density of 0.1 mA cm^–2^ for 1 h for a plating/stripping step. The cell was assembled by stacking two Li discs using an identical method as discussed above.

For anodic/cathodic stability tests, SPC-based electrodes were first placed on the top of the CP-EM (SPC-Al or SPC-Cu/CP-EM) and a portion cut into 10 mm-diameter discs (geometric area = 0.78 cm^2^) using a stainless-steel punch. Another portion of the Li/CP-EM assembly described above was cut into 14 mm-diameter discs (geometric area = 1.54 cm^2^), a PET O-ring (outer diameter = 16 mm, inner diameter = 10 mm) was placed on its top surface, and the 10 mm-diameter assembly (carbon-based electrode/CP-EM) was centered by fitting precisely within the 10 mm inner area of the O-ring. All cells were assembled in an Ar-filled glovebox (MBraun, O_2_ and H_2_O contents <1 ppm). The cathodic stability was examined by cyclic voltammetry (CV) on a Li|CP-EM|SPC-Cu cell, scanning the potential from the open-circuit voltage (OCV) to 0.01 V vs Li^+^/Li and then repeating in the potential range of 0.01–2.0 V vs the Li^+^/Li cell for 10 cycles. An additional cell, as above, however using a bare Cu disk as the working electrode, was assembled for comparison. The anodic stability was determined by linear sweep voltammetry (LSV) from the OCV to 5.0 V vs Li^+^/Li using a Li|CP-EM|SPC-Al cell. The carbon-based electrode was selected for evaluating electrochemical stability since carbon material can represent the actual electrode environment in practical lithium-ion batteries. Both measurements were conducted with the scan rate of 0.1 mV s^–1^ at 70 °C. All cells for electrochemical measurements were preheated at 70 °C for 24 h before testing, to improve the interfacial contact between the electrode and electrolyte. Figure S2 (Supporting Information) displays an illustration of cell preparation for better understanding.

### 2.7 Battery Testing of the CP-EM in Half-Cells and Full Cells

The CP-EM was evaluated for battery application using the LFP cathode in a MEA setup (LFP-MEA) as well as the Li_*y*_SiO_*x*_C anode, both in Li half-cells and Li-ion full cells as described below. The LFP-MEA was prepared by casting the CP-EM as reported above, with the same UV exposure time, thickness gap of doctor blade, and drying process, instead directly on the LFP electrode tape (see photographic image in Figure S3 (Supporting Information). After casting, the CP-EM formed a thin solid electrolyte overlayer of approximately 20 μm as measured by a thickness gauge on top of the LFP cathode. Scanning electron microscopy (SEM; JEOL JSM-6000 Plus, JEOL Ltd., Japan) was employed to examine the cross-sectional morphology of LFP-MEA. The cross-sectional SEM image in Figure S4 (Supporting Information) confirmed the infiltration of the CP-EM into the porous LFP layer, as well as the membrane thickness measured previously. Afterward, the LFP-MEA was punched into an 8 mm-diameter disk (geometric area = 0.5 cm^2^) and stacked on a 12 mm laminated Li/CP-EM with the presence of a PET O-ring (outer diameter = 16 mm, inner diameter = 8 mm). Then, various stackings as above were assembled into coin cells (CR2016, MTI Corp.), which were galvanostatically cycled with voltage ranging from 2.5 to 3.9 V, temperatures from 70 to 90 °C, and various C-rates (1C = 170 mA g^–1^). The same cell setup has also been subjected to CV at 85 °C by scanning the potential from 2.5 to 4.1 V vs Li^+^/Li with a rate of 0.1 mV s^–1^, collecting EIS at the OCV and upon cycles. For the SiO_*x*_C anode study, the MEA setup was avoided by the necessity of anode prelithiation to prevent irreversible Li^+^ consumption, possibly from the LFP cathode (finite Li source), during the formation of the solid electrolyte interphase (SEI) in the first cycle, as suggested in the previous paper.^31^ The prelithiation process was conducted in an Ar-filled glovebox by placing a weighed 8 mm-diameter SiO_*x*_C disk on a Li-metal foil wet with LP30 under a pressure of 2 kg cm^−2^ for either 2 h (half-cell) or 2.5 h (full cell) and subsequent drying for a few minutes at 25 °C to remove the residual solvent. This process yielded the activated SiO_*x*_C anode, hereafter denoted as Li_*y*_SiO_*x*_C, where *y* represents the sacrificial lithium content required to compensate for the specific first-cycle irreversible capacity of the SiO_*x*_C anode in the CP-EM. The prelithiation of the anode was conducted using a liquid electrolyte (LP30) primarily to ensure optimal interfacial contact and uniform lithium distribution throughout the silicon oxide-carbon structure during the process. Indeed, the liquid electrolyte provided sufficient wetting and fast ion transport kinetics into the depth of the porous structure of the electrode during the direct prelithiation step and ensured the formation of a stable and uniform SEI, preventing the first-cycle irreversible capacity loss as reported in our previous study.^31^ The Li_*y*_SiO_*x*_ disk (geometric area = 0.5 cm^2^) was stacked on a 12 mm laminated Li/CP-EM with a PET O-ring (outer diameter = 16 mm, inner diameter = 8 mm), assembled into coin cells (CR2016, MTI Corp.), and tested by galvanostatic cycling between 0.01 and 2.0 V at 70 °C with a constant current of 0.1 mA cm^–2^. Finally, Li_*y*_SiO_*x*_C|LFP-MEA full cells were assembled with a negative-to-positive (N/P) capacity ratio of 1.16, calculated considering the reversible values for Li_*y*_SiO_*x*_C and LFP-MEA in half-cells upon few activation cycles. For full-cell assembly, the Li_*y*_SiO_*x*_ anode prelithiated for 2.5 h and the LFP-MEA, both with a diameter of 8 mm, were coupled and held into a PET O-ring spacer to prevent edge-short-circuiting of the thin membrane during cell crimping as previously described. The cell was cycled at various C-rates (based on the theoretical capacity of LFP) within a voltage range of 1.5–4.0 V at a temperature of 70 °C. A Li_*y*_SiO_*x*_C|LFP full-cell has been assembled using a conventional LP30 electrolyte and cycled at a C/5 rate at 25 °C within the same voltage limits for comparison.

## 3 Results and Discussion

### 3.1 Chemical Structure and Molecular Weight Distribution of Neat Copolymers

To achieve an optimized balance between ionic conductivity and mechanical integrity, the CP-EM includes two P­(EC/EO/AGEC/AGEO) copolymers designed with complementary structural characteristics (see paragraph 2.4). The first copolymer (neat_140k_) is prepared with EO/AGE = 10:1 (considering both AGEC and AGEO) to get a higher molecular weight but a lower fraction of cross-linkable AGE units, for increasing the viscosity of the electrolyte precursor while minimizing hindering ion mobility. Conversely, the second copolymer (neat_28k_) is prepared with EO/AGE = 2:1 to achieve a lower molecular weight but a higher ratio of cross-linkable AGE units, as an increased content of these moieties has been previously found to facilitate LiFSI dissolution at high salt concentrations.^28^ Combining these copolymers with a 50:50 weight ratio by the blending method described above is therefore expected to synergistically yield a matrix with efficient salt dissolution, optimal processability, adequate ion transport, and robust structure. Figure 1 depicts the ^1^H NMR spectra (panel a) and the molecular weight distribution achieved by SEC (panel b) of the copolymers before cross-linking into the CP-EM. The characteristic ^1^H NMR signals are assigned to individual protons in specific linkages according to the findings of a previous paper on an analogous electrolyte.^28^ In brief, overlapping signals observed at 4.1–4.4 ppm are attributed to methylene protons in the carbonate backbone (–CH_2_–C­(–O)– and –C­(–O)–CH_2_– unit adjacent to ether linkages), as well as methylene and methine protons from AGE-derived carbonate units (–CH_2_–CH–C­(–O)–). The small peaks observed at 4.9–5.1 ppm correspond to the methylene and methine protons in the AGE-derived carbonate unit (–C­(–O)–CH_2_–CH– and –CH_2_–CH–C­(–O)–), adjacent to the AGE-derived ether linkages. The broad overlapping resonance observed at 3.4–3.9 ppm is assigned to protons in ether units, originating from both EO and AGE-derived ether units. The assignment of these peaks includes ether protons from EO units in the main chain (–CH_2_–O– and –O–CH_2_– adjacent to carbonate units) and AGE-derived ether units, comprising CH_2_ and CH groups (–CH_2_–CH–O– and –CH–CH_2_–O–) connected through both AGE-derived carbonate and ether backbones, as well as the methylene proton in the ether groups (–CH_2_–O–) linking the side chain ether units to the main chain. The resonance at approximately 4.0 ppm corresponds to the allylic protons (–O–CH_2_–CH=CH_2_) adjacent to the ether linkage and the terminal C=C bond in the side chain. Meanwhile, the peaks at 5.9 and 5.2 ppm are assigned to the protons on the internal (–CH=CH_2_) and terminal sides (–CH=CH_2_) of the double bond, respectively. The quantitative unit composition of each copolymer (EC/EO/AGEC/AGEO) is determined by ^1^H NMR spectra curve fitting, using the Lorentzian function according to a calculation method and equations reported in our previous study.^40^ Table 1 summarizes the resulting compositional ratios, as well as the molecular weight distributions of the copolymers analyzed via SEC, as presented in Figure 1b. Although both copolymers are synthesized under analogous conditions (catalyst ratio, temperature, time, stirring speed, and CO_2_ pressure), their SEC profiles differ significantly. The copolymer with lower AGE content (EO/AGE = 10:1) exhibits a monomodal distribution with a relatively narrow dispersity (*D̵* = 1.4) with *M*_*n*_ = 14 × 10^4^ (neat_140k_), indicating a well-controlled polymerization process. Instead, increasing the AGE ratio (EO/AGE = 2:1) results in a shift toward lower molecular weight with *M*_*n*_ = 2.8 × 10^4^ (neat_28k_), accompanied by a significantly broader and bimodal distribution (*D̵* = 3.7). The broadened and bimodal nature of the Neat_28k_ curve suggests the possibility of side reactions likely involving the allyl moieties during polymerization. Such phenomena can potentially lead to a wider heterogeneity in chain lengths and earlier termination of polymer growth.

**Figure 1:**
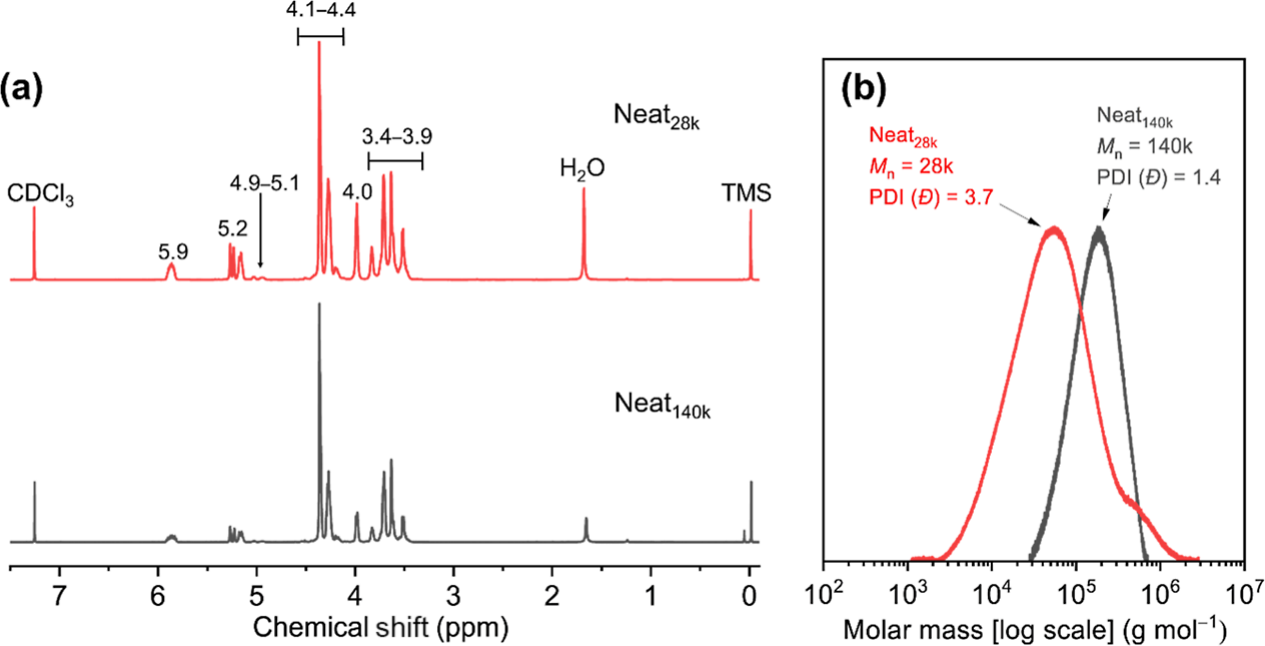
(a) ^1^H NMR spectra of the copolymer before cross-linking (Neat_28k_ and Neat_140k_) in CDCl_3_. (b) Molar mass distributions (GPC traces) of Neat_140k_ and Neat_28k_ determined relatively to the polystyrene standards.

**Table 1:** ^a^Copolymer Composition Determined by ^1^H NMR Spectroscopy Using the Integrals of Characteristic Units for Copolymer before Crosslinking and ^b^Molecular Weight Characteristics Obtained by SEC in CHCl_3_ Using Narrow-Dispersity Polystyrene Standards

copolymer	monomer unit^a^ (%)				*M*_*n*_^b^	*M*_w_^b^	PDI^b^
	EC	EO	AGEC	AGEO			
neat_140k_	54	29	5	12	14 × 10^4^	19 × 10^4^	1.4
neat_28k_	41	28	14	17	2.8 × 10^4^	10 × 10^4^	3.7

### 3.2 FT-IR Analysis of the CP-EM

After confirming the copolymer composition and molecular weight, the two neat substrates are blended with LiFSI and the photoinitiator in ACN to obtain CP-EMs as reported above. Although our previous study for analogue electrolyte indicated that 15 min of UV irradiation in an Ar-filled glovebox is sufficient to obtain a self-standing membrane,^40^ the exposure time is herein extended to 30 min since the CP-EM is advantageously achieved in ambient conditions, where the presence of oxygen can scavenge free radicals and retard the cross-linking reaction.^41^ The successful UV-induced cross-linking of the polymer matrix under ambient conditions is confirmed by FT-IR spectroscopy. The FT-IR spectra in the 1800–1620 cm^−1^ region, and the components magnified in the 1660–1620 cm^–1^ interval shown in Figure S5 (Supporting Information) reveal a reduction in the intensity of the characteristic allyl C= C stretching peak at 1645 cm^–1^ of the CP (without salt) and the CP-EM compared to neat polymer,^42^ thus suggesting an efficient cross-linking. On the other hand, a copolymer conversion ratio below 100% cannot be excluded since more accurate determination may require highly specialized techniques, such as solid-state NMR, which are designated for future work. To investigate the dissociation of LiFSI and its interaction with the copolymer matrix, the FT-IR spectra of the pristine cross-linked polymer (CP) and the electrolyte membrane (CP-EM with 140 mol % LiFSI) are compared in the 1800–1670 cm^–1^ and 900–800 cm^–1^ intervals as reported in Figure 2. The pristine CP (Figure 2a) exhibits the characteristic stretching vibration of free carbonyl groups (C=O) at 1740 cm^–1^ ^23^ that noticeably downshifts to approximately 1720 cm^–1^ upon the incorporation of 140 mol % LiFSI. This shift indicates a weakening of the C=O, driven by the coordination between the carbonyl oxygen and the Li^+^ (C=O···Li^+^). Furthermore, peak deconvolution reveals that approximately 80% of the carbonyl groups in the CP-EM actively participate in this Li^+^ solvation. The significant downshift of the C=O band, and the relatively small changes in the ether (C–O–C) stretching^27^ detected from the FT-IR spectra of CP and CP-EM within 1500 and 900 cm^–1^ in Figure S6 (Supporting Information), suggest the Li^+^ ions predominantly coordinate with the highly polar carbonyl groups of the carbonate segments rather than the ether oxygens.^27^ Figure S6 also evidences the vibrations related to the FSI^–^ anion promoted by salt inclusion into the CP, with dominant peaks at around 1170 cm^–1^ and 1115 cm^–1^ reportedly due to the stretching vibration modes (ν_SO2_ and ν_Li‑SO2_) of the FSI anion.^43^ The FT-IR spectra of Figure 2b collected in the wavenumber range of 900–800 cm^–1^ reveal for the CP-EM the characteristic FSI^–^ peak (ν_S‑F_) at 840 cm^–1^ (top spectrum), which is absent for the salt-free CP (bottom spectrum). It is worth mentioning that the above-mentioned FSI^–^ peak appears herein with a shift of 20 cm^–1^ to a higher wavenumber compared to the copolymer previously studied, adopting a lower salt amount (i.e., 10 mol % of LiFSI rather than 140%),^27^ mainly due to ion-aggregation effects. On the other hand, the observed peaks of the ν_Li‑SO2_ at 1115 cm^–1^ and ν_S‑F_ at 840 cm^–1^ indicate that the LiFSI salt is not fully dissociated into free ions. Instead, a significant fraction of the FSI^–^ anions is directly coordinated to Li^+^ with formation of contact ion pairs (CIPs) or ionic aggregates.

**Figure 2:**
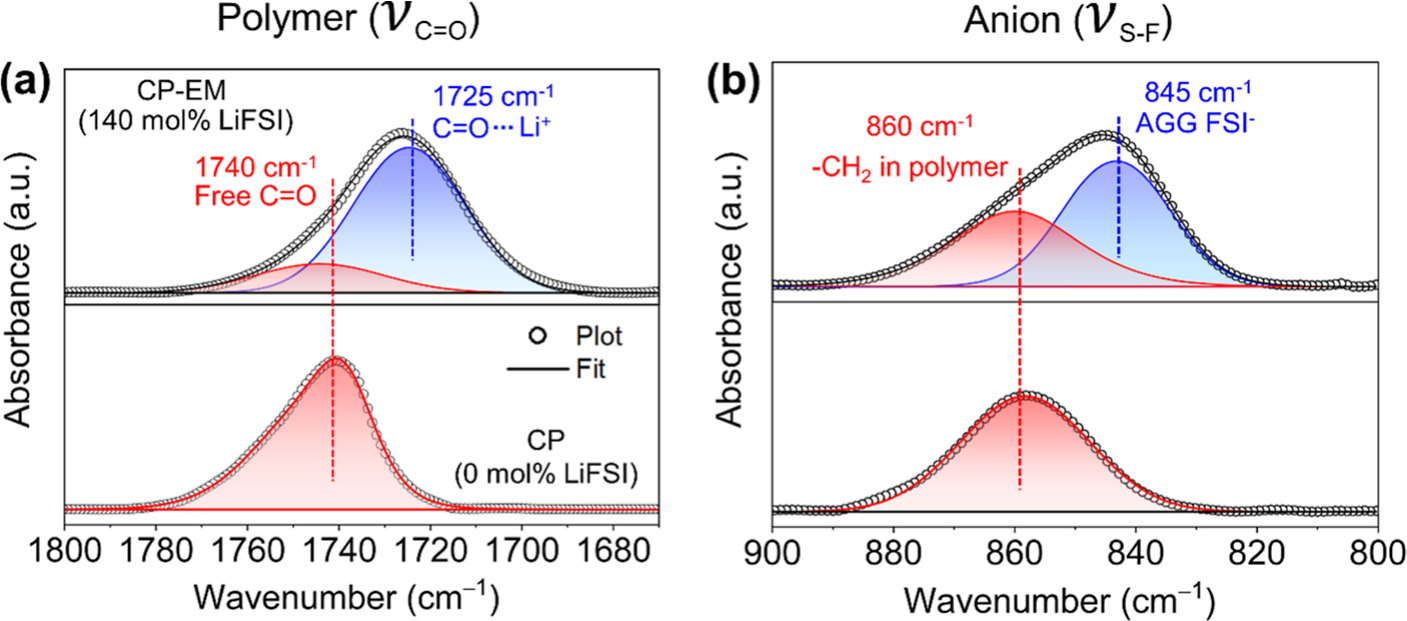
Peak deconvolution of FT-IR spectra with the stretching vibration of (a) C=O (υ_C_=_O_) of the copolymer in the wavenumber range of 1800–1670 cm^–1^ and (b) S–F (υ_S‑F_) of the FSI anion in the wavenumber range of 900–800 cm^–1^ for the CP (bottom) and the CP-EM (top).

### 3.3 Thermal, Mechanical, and Electrochemical Properties of the CP-EM

After the drying process, the CP-EM is held in an Ar-filled glovebox for at least 24 h before use (see Experimental Section), which is demonstrated to be an efficient procedure to equilibrate the polymer and uniform the surface by Ar gas physisorption. This phenomenon is evidenced by TGA under N_2_ flow of CP-EM before drying, with isotherms cyclically held at 70 °C (expected gas desorption) and 30 °C (expected gas absorption). The result in Figure S7 (Supporting Information) confirms the above hypothesized behavior since the sample weight decreases by 3% by holding the initial temperature at 70 °C as the residual solvents (mainly ACN and H_2_O) are removed, while it increases back by about 2% when the CP-EM is held at 30 °C due to N_2_ gas absorption. The subsequent TGA cycles reveal similar trends, respectively, showing repeated weight decrease and increase by raising or lowering the temperature as N_2_ adsorbs and desorbs, however, with a lower magnitude compared to the first thermal cycle. Thermal stability of the CP-EM is particularly relevant since the carbonate units in the polymer backbone may suffer from backbiting reactions at elevated temperatures.^44,45^ Therefore, TGA with corresponding DTG is performed in Figure 3 from 25 to 800 °C on a dry CP-EM (panel a), for comparison on a cross-linked polymer without salt (CP, panel b), and on the conducting salt (LiFSI, panel c). The thermal profile of the CP-EM in Figure 3a shows weight loss less than 1% in the temperature range from 60 to 120 °C, which corresponds to the removal of ACN or moisture traces. The minor mass change indicates that the CP-EM is effectively dried after the preparation process conducted under ambient conditions (see Experimental Section). Figure 3a shows for the CP-EM the first consistent weight loss of about 70% in the temperature range from 200 °C to about 400 °C, revealing a DTG signature composed of a shoulder at 175 °C, and a series of peaks centered at about 195, 235, and 285 °C, with a further shoulder at 315 °C. This behavior may be explained by considering the TGA/DTG curve of the components, i.e., the CP without salt and the LiFSI salt itself. Indeed, the TGA of CP in Figure 3b reveals an initial weight loss of 5% ascribed to absorbed water below 100 °C and a main weight loss likely due to carbonate and ether segments decomposition from 220 to 420 °C, with DTG peaks centered at approximately 280 and 360 °C, and a shoulder at 410 °C.^26^ Furthermore, Figure 3c indicates that the LiFSI salt loses crystallization water below 200 °C through various steps and decomposes above this temperature with DTG peaks and shoulders at 230, 290, 330, and 395 °C according to the literature.^46^ These results suggest that polymer segments and salt are homogeneously incorporated within the cross-linked network, since the DTG in Figure 3a reflects the combination of the ones in Figure 3b,c, with predominance of the salt signature indicating degradation largely governed by the latter within the polymer matrix. On the other hand, the CP-EM shows decreased decomposition temperatures compared to CP, thus suggesting that the incorporation of 140 mol % LiFSI significantly alters the polymer degradation pathway. Indeed, the dissociated ions (Li^+^ and FSI^–^) can weaken the thermal activation of ether (C–O) and carbonyl (C=O) bonds, similarly to what is observed in conventional PEO-based electrolytes,^47^ while S–N and S–O bonds in FSI can undergo easy scission.^48^ The full degradation, leading to LiF formation with SO_2_ and NO_2_ evolution from the dissociated salt,^46,49^ is observed at about 660–720 °C for the CP-EM with DTG centered at 700 °C (Figure 3a). Instead, the same process is retarded possibly to a temperature higher than 800 °C for the bare LiFSI salt (Figure 3c). To further characterize the thermal properties and determine the glass transition temperature (*T*_g_), DSC is conducted over a temperature range from −80 to 150 °C with outcomes reported in Figure S8 (Supporting Information). The *T*_g_ of the CP-EM is identified to be approximately −31 °C during the second heating scan and remains almost the same by the subsequent cycle. On the other hand, the DSC results of poly­(alkylene carbonate) homopolymer electrolytes can typically show a significant shift in *T*_g_, attributed to degradation induced by electrophilic activation from the lithium salt at elevated temperatures.^50^ This consistent thermal behavior indicates that the CP-EM possesses relevant thermal stability without undergoing degradation at elevated temperatures up to 150 °C. In addition to the thermal stability, the stress–strain curve in Figure S9 and related mechanical properties in Table S1 (Supporting Information) reveal for the CP-EM an elastomeric-like behavior, with the estimated maximum stress of 0.53 MPa, Young’s modulus of 0.20 MPa, and elongation at break of 210%. Overall, these findings indicate for the CP-EM a low *T*_g_ which facilitates ion mobility via the segmental motion of the polymer chains, while simultaneously holding sufficient mechanical robustness and thermal stability at elevated temperatures.

**Figure 3:**
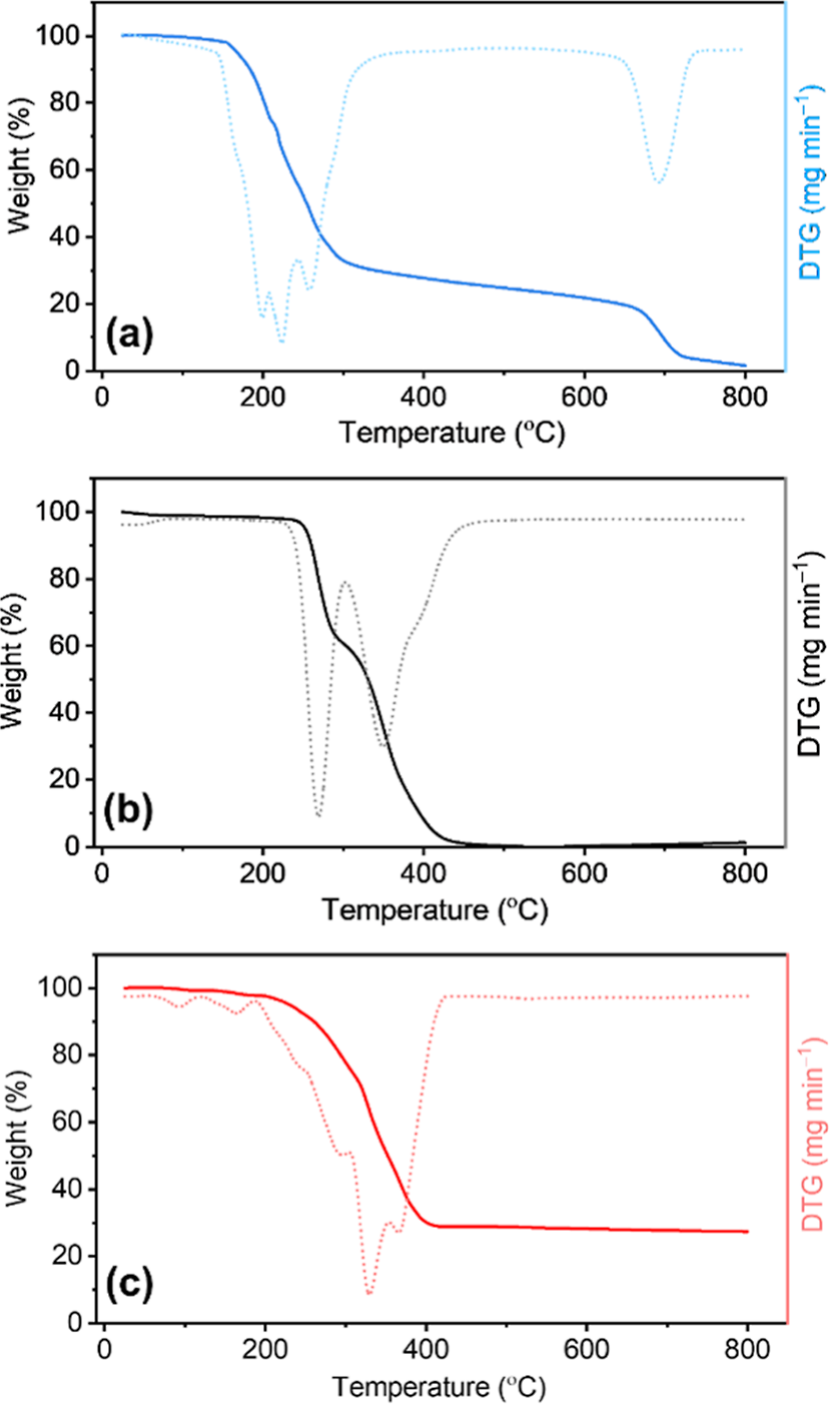
TGA (solid lines) and corresponding DTG curves (dot lines) of the (a) CP-EM, (b) CP without salt, and (c) LiFSI salt performed in the 25–800 °C temperature range under N_2_ flow of 50 mL min^−1^, with a heating rate of 5 °C min^–1^.

Figure 4 summarizes crucial electrochemical characteristics of the CP-EM for battery application. The CP-EM ionic conductivity (Figure 4a), obtained by fitting the Nyquist plot of Figure S10 with the equivalent circuits of Table S2 (Supporting Information), ranges from 1.1 × 10^–4^ S cm^–1^ at 98 °C to 2.7 × 10^–7^ S cm^–1^ at 30 °C. The low room-temperature ionic conductivity of ∼3 × 10^–7^ S/cm restricts the application range of our medium to one typical of the SPEs, e.g., in practical devices such as stationary energy storage systems (ESS) and high-temperature industrial electronics, where thermal stability and safety offer distinct advantages over conventional electrolytes. Furthermore, the conductivity trends are properly described by Vogel–Tammann–Fulcher (VTF) behavior, with coupled ion-segmental polymer chain transport rather than a simple thermally activated ion motion process.^51,52^ Therefore, the pseudoactivation energy (*E*_a_) for ion transport is estimated using the VTF eq 3 as follows^53^

3 $$\sigma =\sigma_{\infty }\exp\left(-\frac{E_{a}}{k_{B}\left(T-T_{0}\right)}\right)$$

where *T*_0_ (K) is the temperature for zero configurational entropy, which is typically ∼30 K lower than the glass transition temperature (*T*_g_) of the electrolyte, σ_∞_ (S cm^–1^) is the ionic conductivity at infinite temperature, and *k*_B_ is the Boltzmann constant (8.62 × 10^−5^ eV K^–1^), as indicated in Table S3 in Supporting Information (a, b, and c stand for σ_∞_, (*E*_a_/*T*_0_)/*k*_B_, and 1000/*T*_0_, respectively).^53^ The *E*_a_ of the CP-EM is calculated to be 0.110 eV, which is a value slightly lower than the one achieved in a previous study using a smaller amount of cross-linking units as well as concentration of salt (at 80 mol % of LiFSI, *E*_a_ = 0.154 eV).^54^ This suggests that high salt concentration provides less activation energy for Li^+^ migration in the copolymer matrix, while balanced cross-linking units improve mechanical robustness. In addition, the estimated *E*_a_ value appears significantly lower than that of the typical PEO-based solid polymer electrolyte, such as the one reported in the literature with PEO of *M*_w_ = 6 × 10^5^ g mol^–1^ and LiFSI at a 2:1 mass ratio, which exhibited an activation energy of approximately 0.68 eV.^55^ It is well-known that the crystallization of PEO-based SPEs limits ion mobility due to the strong coordination between oxygen atoms and Li^+^.^56,57^ The lower *E*_a_ value therefore indicates that the amorphous CP-EM matrix provides a favorable environment for Li^+^ transport, thus allowing an easier activation of ion motion. These findings consistent with the low *T*_g_ value of the CP-EM, which provides high segmental mobility of the amorphous cross-linked network and facilitates the efficient Li-ion transport observed in the system. Based on these findings, it is worth mentioning that an adequate balance of the copolymer structure and salt concentration is a crucial step toward the achievement of targeted properties. To evaluate the mobility of Li^+^ ions in the CP-EM system, the *t* ^+^ is determined in Figure 4b by chronoamperometry in combination with EIS using the Li|CP-EM|Li symmetric cell configuration at 70 °C. A direct current bias of 30 mV is applied for 1.5 h (see Experimental Section), which is a low voltage for preventing undesired side reactions during polarization, with a sufficient time for ensuring ion diffusion limit at the steady-state condition. The corresponding Nyquist plots recorded before and after cell polarization (inset of Figure 4b) are analyzed with NLLS as summarized in Table 2, and the *t* ^+^ subsequently is calculated to be 0.61 using and the Bruce–Vincent–Evans eq 2.^38^ The *t* ^+^ value of the CP-EM is only slightly lower than that of the salt-concentrated non-cross-linked P­(EC/EO)-SPE (*t* ^+^ = 0.66), with a difference of merely 0.06.^27^ This minor reduction can be attributed to the trade-off between ionic mobility and mechanical reinforcement in the cross-linked network, which may provide a higher stability at elevated temperatures. It should be mentioned that the *t* ^+^ for the CP-EM moderately exceeds that of conventional liquid electrolytes based on LiPF_6_ or LiFSI^58–60^ and significantly overcomes that of the PEO-based SPEs either with LiFSI or LiTFSI, which is typically less than 0.4.^61,62^ This remarkable performance is particularly striking considering the high salt content (140 mol % or 75 wt % relative to the total salt and polymer), which can possibly increase ion pairing and reduce Li^+^ mobility. The result suggests that the polymer architecture and blending strategy actually promote the Li^+^ transport in the CP-EM even within high salt concentration. It is worth mentioning that pulsed-field gradient (PFG) NMR measurements can actually allow the determination of the self-diffusion coefficients of Li^+^ and FSI^–^ and provide indeed a deeper molecular-level insight into the ion transport mechanisms and the specific influence of the polymer architecture on ion coordination. On the other hand, this technique has already shed a relevant light on important electrolytes, even including those based on long- and short-chain glymes.^63–65^ However, the complexity and the extensive nature of PFG-NMR may require a dedicated study and *ad hoc* investigation focused specifically on the ion–polymer interactions and ion diffusion of various cross-linked systems. The compatibility of lithium metal with the electrolyte is another crucial factor in determining the overall performance of the CP-EM. Therefore, this parameter is examined by monitoring the interfacial resistance of Li|CP-EM|Li symmetric cells aged at 70 °C for 168 h (7 days) using the EIS technique. The corresponding Nyquist plots in Figure S11 (Supporting Information) are fitted to an equivalent circuit to evaluate the bulk resistance (*R*_b_) and interfacial resistance (*R*_*i*_) (details of the fitting procedure are provided in the Experimental Section). The obtained resistance values are summarized in Table S4 and plotted as a function of time in Figure 4c. Initially, both *R*_b_ and *R*_*i*_ exhibit relatively high values on the order of 10^4^ Ω at room temperature, significantly decreasing to 636 Ω and 165 Ω upon heating the cell at 70 °C for 1 h due to the increase in segmental motion of the polymer and of the Li^+^ ions promoted by thermal activation. With prolonged aging, *R*_b_ further decreases and eventually stabilizes after 168 h, whereas *R*_*i*_ gradually increases. The increase of *R*_*i*_ can be attributed to the progressive formation and thickening of the SEI layer at the Li/CP-EM interphase, which can partially hinder the charge transfer.^66,67^ Furthermore, the decrease of *R*_b_ over time is consistent with this phenomenon since the formed SEI can reduce the effective distance between the electrode and electrolyte (i.e., *l* variable in eq 1), lowering the bulk resistance across the cell. A wide electrochemical stability window (ESW) of solid electrolytes is certainly crucial for achieving suitable performance in a practical battery. Therefore, the ESW of the CP-EM is evaluated using CV in the Li|CP-EM|SPC-Cu cell for the cathodic region and LSV in Li|CP-EM|SPC-Al cell for the anodic one, as presented in Figure 4d. The first CV run for checking the cathodic stability reveals a reduction peak centered at approximately 0.9 V vs Li^+^/Li, likely attributed to the decomposition of the LiFSI salt to form the SEI layer in the electrode surface. This behavior has been previously reported for the 1 M LiFSI liquid electrolyte, which showed reduction at ∼0.8 V in a Li|graphite cell at 30 °C.^68^ The slight shift of 0.1 V can be ascribed to several factors, including the characteristic solvation structure of LiFSI within the cross-linked polymer matrix, the specific salt concentration, and the temperature. Following the initial reduction scan, repeated CV between 0.01 and 2.0 V vs Li^+^/Li shows an electrochemical trend with anodic peaks at 0.15 and 1.05 V vs Li^+^/Li, reversed into cathodic ones at 0.8 and 0.05 V vs Li^+^/Li, respectively, due to (de)­insertion and possible stripping/deposition of Li^+^ ions into the structure of the SPC.^30^ Additional CV for cathodic stability evaluation is performed in Figure S12 (Supporting Information) on a Li/CP-EM/Cu cell and compared with that of the Li/CP-EM/SPC-Cu cell of Figure 4d. Interestingly, CV using the bare Cu shows relevant noise with various current spikes of very modest intensity (circled in Figure S12). Furthermore, the same CV reveals an electrolyte reduction peak shifted up to 1.5–2.0 V vs Li^+^/Li rather than 1 V observed for SPC and with a lower intensity. The above noise may be ascribed to nonoptimized contact between the flat Cu and the CP-EM, that can trigger microshort circuits or parasitic signals. Instead, the potential shift and peak intensity reduction may be associated with the different chemical environment of CP-EM/Cu compared to CP-EM/SPC. In addition, the CV profile of bare Cu does not show anodic and cathodic peaks related to the (de)­insertion/deposition of Li at low potentials such as those observed using SPC. The anodic stability is subsequently evaluated by LSV from OCV to 5.0 V vs Li^+^/Li. The CP-EM exhibits a two-step oxidation process, with a first onset at approximately 4.1 V vs Li^+^/Li where the current density reaches 10 μA cm^–2^ and a second one at 4.5 V vs Li^+^/Li with a current higher than 20 μA cm^–2^ upon further increasing the potential. We attribute the first current onset at 4.1 V vs Li^+^/Li to the oxidation of ether-rich segments predominantly originating from the low-cross-linked copolymer (CP_140k_),^69^ while the second one at 4.5 V vs Li^+^/Li to the more stable rigid carbonate/cross-linked domains, as the incorporation of the AGE unit can suppress the oxidation at a lower potential.^28^ Nevertheless, these findings demonstrate that the CP-EM possesses adequate ESW for application as a solid electrolyte with 3–4 V-class olivine cathodes in a lithium-ion battery.^70,71^ The long-term Li deposition/stripping behavior of the CP-EM electrolyte is investigated using a Li|CP-EM|Li symmetric cell at 70 °C within 1 h of charge/discharge under a constant current density of 0.1 mA cm^–2^, as illustrated in Figure 4e. The cell exhibits a moderately stable voltage profile, with a nearly constant overpotential of approximately 40 mV for over 150 h, and a slight increase to 45 mV at the end of the test after 200 h. Such a trend demonstrates that the cross-linked polymer matrix provides the formation of a stable interfacial layer between the Li metal and the CP-EM, even though at a relatively high temperature (70 °C). Despite an overpotential mostly constant across the 200 h, occasional voltage spikes with transient drop or increase occur during cycling, mainly due to soft-short circuit phenomena.^72^ The soft-short circuit represents a localized and small electrical contact between the two Li electrodes separated by the CP-EM, as a microdendrite grows on a defective SEI, reaches the opposing electrode upon penetrating the electrolyte, and subsequently dissolves, allowing the stripping/deposition process to proceed.^73^ The recovery of initial polarization (∼0.45 mV) upon soft-short circuits suggests that the CP-EM can potentially tolerate microdendritic Li growth, holding a moderate interfacial resistance during repeated cycling. This behavior can be attributed to the synergy of the two copolymers, blended at high salt concentration, in which the low cross-linking unit, together with high EO contents (CP_140k_), supports the ion mobility by the softer segment, while the high cross-linking unit (CP_28k_) reinforces the mechanical integrity of the interphase. These data imply that the polymer matrix has sufficient elasticity and mechanical robustness to prevent dendrite propagation.

**Figure 4:**
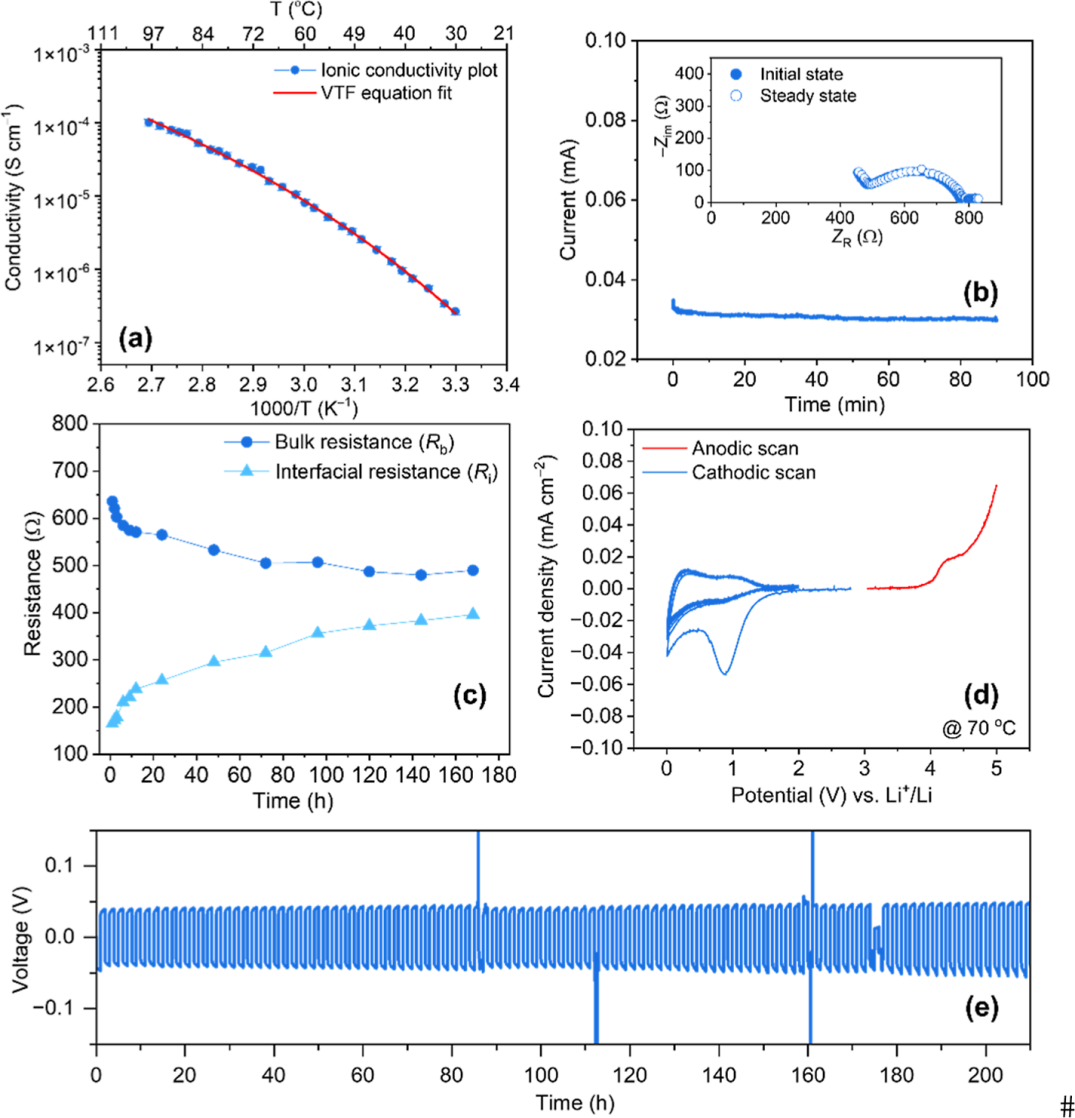
Electrochemical analyses of the CP-EM. (a) Ionic conductivity as a function of temperature; the corresponding Nyquist plots are shown in Figure S10 with resistances in Table S2. (b) Chronoamperometry curve for the determination of *t* ^*+*^ using the Bruce–Vincent–Evans method (eq 2). A DC bias of 30 mV was applied at 70 °C, and impedance spectra (AC amplitude: 10 mV, 500 kHz to 100 mHz) were collected for the Li|CP-EM|Li cell before (initial state) and after polarization (steady state) as reported in the inset. (c) Evaluation of bulk and interfacial resistances upon aging of Li|CP-EM|Li symmetric cells, obtained from impedance measurements in the 500 kHz to 100 mHz frequency range (AC amplitude: 10 mV). The corresponding Nyquist plots and NLLS fitting results are presented in Figure S11 and Table S4. (d) ESW determined by CV in the 0.01–2.0 V vs Li^+^/Li range using Li|CP-EM|SPC-Cu cell and LSV from OCV to 5.0 V vs Li^+^/Li using Li|CP-EM|SPC-Al cell at a scan rate of 0.1 mV s^–1^. (e) Li stripping/deposition profiles of Li|CP-EM|Li symmetric cells cycled at a constant current density of 0.1 mA cm^–2^ with step times of 1 h.

**Table 2:** Direct Currents (DC, *I*_0_, *I*_ss_) and Li/Electrolyte Interfacial Resistances (*R*_0_, *R*_ss_) for Li/CP-EM/Li Symmetric Cells of Figure 4b upon Chronoamperometry with a Voltage of 30 mV Measured at 70 °C for Determining the *t* ^*+*^ Using eq 1

sample	*I*_0_ (mA)	*I*_ss_ (mA)	*R*_0_ (Ω)	*R*_ss_ (Ω)	*t* ^+^
CP-EM	0.035	0.030	543.9	481.1	0.61

### 3.4 Battery Performance of the CP-EM

Despite the suitability of the CP-EM demonstrated by fundamental tests, the proof-of-concept for battery use lies in its performance within a practical environment. For this purpose, an LFP-MEA is prepared and evaluated in lithium cells under multiple operating conditions. To examine the temperature dependence of the performance, the assembled cells are cycled at 70 °C, 80 °C, and 90 °C using a current rate of C/10 (1 C = 170 mA g^–1^), and the corresponding charge/discharge voltage profiles are presented in Figure 5. The cell delivers at 70 °C a charge capacity of 139 mAh g^–1^, and a discharge one of about 143 mAh g^–1^, leading to Coulombic efficiency (%CE) exceeding 100%. The delivered capacity represents ∼87% of the LFP theoretical one, which indicates that the LFP-MEA is well adequate for cell application. Furthermore, an efficiency higher than 100% during the initial cycle is likely attributed to side reduction occurring at the electrode/electrolyte interface during cell discharge, similarly to what was observed in Li|NMC cells using liquid electrolyte^74^ as well as computational models.^75^ After this conditioning process, the CE stabilizes around 99.2%, thus indicating that the charge/discharge reactions settle into a more reversible regime as cycling proceeds, however, with a progressive decrease of the capacity to 127 mAh g^–1^ upon 3 cycles. When the temperature rises to 80 °C at the fourth cycle, the discharge capacity increases back to 135 mAh g^–1^ as promoted by faster ion transport within the electrode/electrolyte interphase. The charge profiles of the fourth and fifth cycles exhibit transient noise due to the above-mentioned soft-shorts, possibly driven by temperature-induced oxidation of the residual initiator or uncross-linked segments in the CP-EM. On the other hand, the cell shows a stable charge/discharge from the sixth to the ninth cycle; however, with a reversible capacity decreasing to 110 mAh g^–1^, possibly due to thickening of the SEI by the above transient side processes, and associated resistance and polarization increase. A further rise in the temperature to 90 °C increases the reversible capacity of 160 mAh g^–1^, which is about 96% of the theoretical one for the LFP.^32^ In addition, the polarization at 90 °C decreases, and the voltage profile flattens, becoming less affected by diffusive phenomena compared to the ones at 70 and 80 °C. This behavior is principally due to a decrease of the cell resistance and fastening of the lithium charge transfer as triggered by the temperature increase.^76,77^

**Figure 5:**
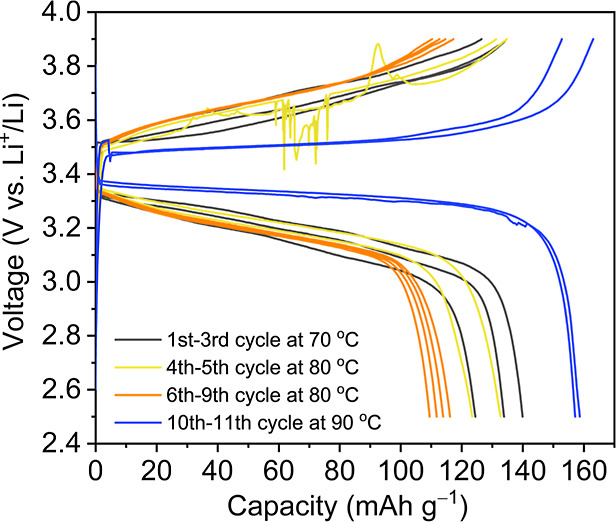
Voltage profiles of Li|CP-EM|LFP-MEA half-cell performed at temperatures progressively increasing from 70 °C to 80 and 90 °C at C/10 rate (1 C = 170 mA g^–1^), within a voltage range of 2.5–3.9 V. LFP mass loading: 6 mg cm^–2^.

Although the cell can relevantly stand up 80 and 90 °C as suggested by Figure 5, the temperature of 70 °C is hereafter selected for subsequent tests of the CP-EM in Li half-cells as reported in Figure 6. Hence, the Li|CP-EM|LFP-MEA cell is evaluated at C/10 at 70 °C, with charge/discharge profiles shown in Figure 6a. The first cycle delivers charge and discharge capacities of 122 and 123 mAh g^–1^, respectively, corresponding to an initial %CE of ∼101%, consistent with the previous cell, in which the first cycle %CE exceeded 100% for the reasons discussed earlier. The related cyclic trend in Figure 6b depicts a gradual decrease of the reversible capacity to reach ∼85 mAh g^–1^ at the 15th cycle due to the above-mentioned side reactions leading to SEI growth with resistance and polarization increase. Subsequently, the cell holds the latter value with only minor fluctuations of the %CE, which remains above 99% upon 30 charge/discharge cycles. The rate capability of the cell is evaluated by increasing the C-rate every five cycles from C/20 to C/10 and C/5. The voltage profiles presented in Figure 6c reveal for the first cycle at C/20 a capacity of ∼140 mAh g^–1^, which gradually decreases to ∼132 mAh g^–1^ upon 5 cycles at the same current, as evidenced by the corresponding trend in Figure 6d. As the C-rate is increased to C/10, the cell displays an expected rise in overvoltage, reflecting more relevant ohmic drop at higher current, and the capacity reaches ∼100 mAh g^–1^. A further increase of the C-rate to C/5 leads to a relevant ohmic drop with a residual capacity of ∼25 mAh g^–1^, thus suggesting the latter current rate as the limiting one for practical cell applications. When the C-rate is subsequently lowered back to C/10 after 15 cycles and C/20 after 20 cycles, the cell recovers discharge capacities of 90 and 125 mAh g^–1^, respectively, corresponding to 92% and 94% of the initial values at the same rates. This behavior highlights the robustness of the CP-MEA under varying current demands, even though the ohmic drop at high currents still needs to be addressed.^76^ A further proof of the anodic stability of the Li|CP-EM|LFP-MEA cell at higher voltage is given by CV and EIS before and after each cycle in Figure S13 (Supporting Information), performed by increasing the charge cutoff to 4.1 V vs Li^+^/Li to evaluate possible interface oxidation over cycling. The CV (Figure S13a) reveals an interphase activation from the first to the fourth cycle, denoted by the increase of the LFP peak intensity around 3.5 V vs Li^+^/Li, as possibly promoted by a progressive electrode wetting. On the other hand, the impedance holds a similar shape and overall value of about 1200 Ω from the OCV to the end of the test (Figure S13b), thus suggesting the absence of a relevant oxidation over 4.1 V vs Li^+^/Li. Before assembling a full Li-ion battery, it is essential to confirm that the electrolyte is compatible with the SiO_*x*_-C composite electrode, which is chosen as the preferential anode with a capacity of about 400 mAh g^–1^ and a retention of 94% for 200 cycles in conventional liquid electrolytes.^31^ Therefore, the CP-EM is hereafter combined with a prelithiated SiO_*x*_C, i.e., Li_*y*_SiO_*x*_ (see details in Experimental Section) in a Li half-cell galvanostatically cycled at 0.1 mA cm^−2^. The related voltage profiles in Figure 6e exhibit an initial OCV of ∼0.5 V, consistent with our prior study of the same anode in carbonate-based electrolytes, in which a lowered OCV indicated the actual prelithiation of SiO_*x*_C.^31^ Upon the progress of the test, the voltage profile depicts the characteristic features of the (de)­lithiation of the Li_y_SiO_*x*_-C electrode, with an electrochemical process occurring through various steps leading to multiple plateaus. Indeed, the region centered around ∼0.5 V is mainly associated with the Li (de)­insertion into the amorphous carbon, whereas the low-voltage one between 0.1 and 0.01 V may reflect possible Li–Si (de)­alloying, or Li-stripping/deposition on the electrode. At the first cycle, the cell delivers a discharge capacity of 332 mAh g^–1^ and a charge of 265 mAh g^–1^, thus leading to a %CE of 80% (Figure 6f). The same efficiency is observed in the second cycle, however, with the discharge and charge capacity increased up to 370 and 300 mAh g^–1^, respectively. This relatively low efficiency and the subsequent increase of capacity are expected by a structural organization of the SiO_*x*_C electrode during the initial cycles, as well as by side reductive reactions of the CP-EM leading to the consolidation of the SEI.^31^ Afterward, the %CE increases and stabilizes to reach nearly 95% at the 10th cycle, with a reversible capacity of ∼295 mAh g^–1^. Despite the effectiveness of the prelithiation process, which increases the initial efficiency from the typical value of 40% of the native anode^31^ to over 80%, these data suggest the necessity for a better tuning of the activation time and conditions to ensure a higher efficiency and avoid capacity loss in the specific Li-ion polymer cell used for the CP-EM and Li_*y*_SiO_*x*_C.

**Figure 6:**
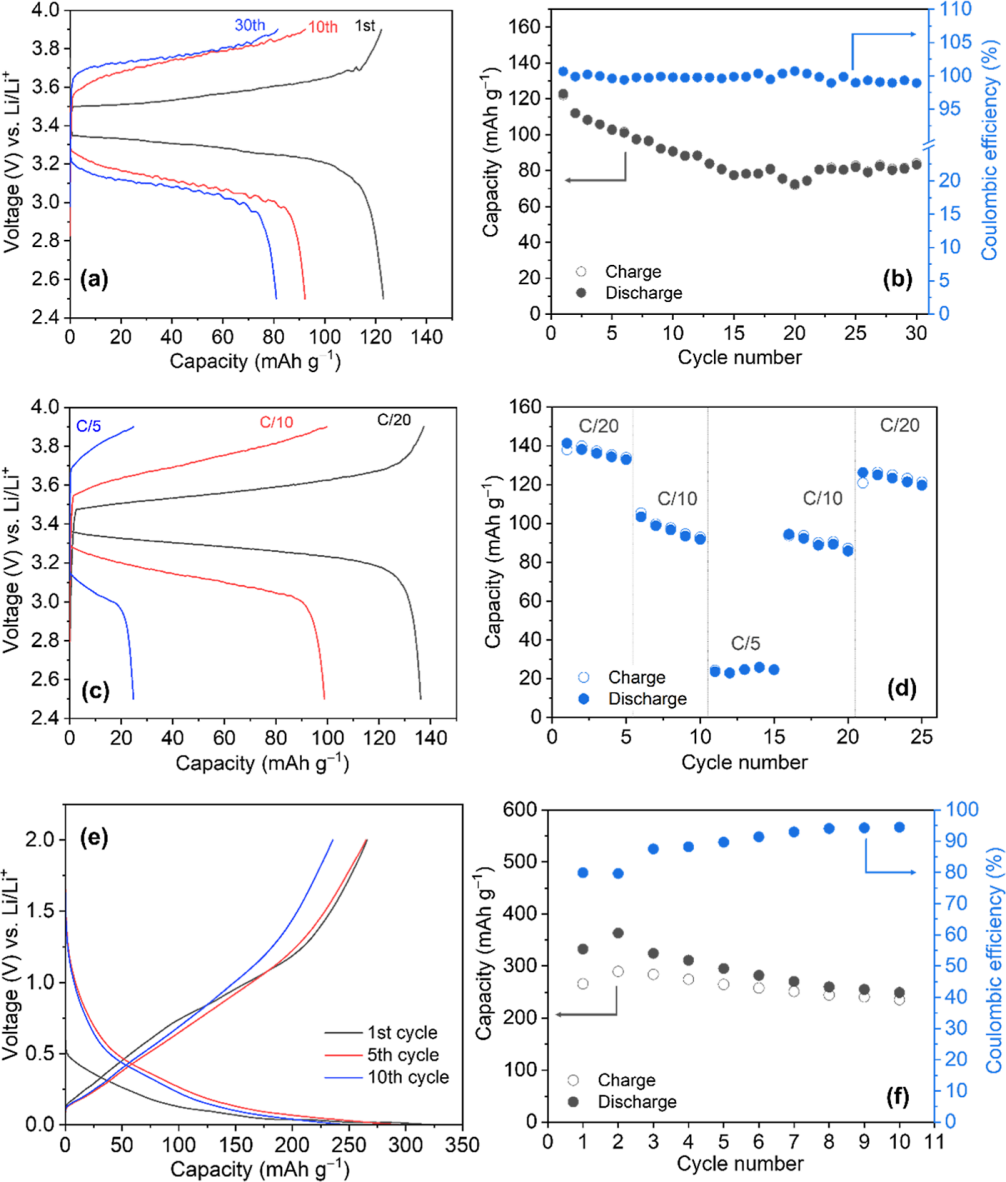
Application of the CP-EM as an electrolyte in a lithium battery. (a,b) Galvanostatic performance of a Li|CP-EM|LFP-MEA half-cell at C/10 in terms of (a) selected charge/discharge voltage profiles and (b) cycling trend (%CE in right-*y* axis). (c,d) Rate capability of a Li|CP-EM|LFP-MEA at C/20, C/10, and C/5 in terms of (c) selected charge/discharge voltage profiles, and (d) cycling trend. 1 C = 170 mA g^–1^, LFP mass loading = 6 mg cm^–2^. (e,f) Galvanostatic performance of a Li|CP-EM|Li_*y*_SiO_*x*_C half-cell at a current density of 0.1 mA cm^–2^ in terms of (e) selected discharge/charge voltage profiles and (f) cycling trend (%CE in right-*y* axis). Li_*y*_SiO_*x*_C mass loading 3.2 mg cm^–2^. Prelithiation time: 2 h. All tests performed at 70 °C.

Taken together, the results of Figure 6 demonstrate that the CP-EM is simultaneously compatible with the olivine LFP cathode in the MEA setup and the SiO_*x*_-based anode, highlighting its suitability as an electrolyte capable of enabling the all-solid Li-ion Li_*y*_SiO_*x*_C|LFP-MEA full cell.

The galvanostatic cycling performances of the Li_*y*_SiO_*x*_C|LFP-MEA Li-ion battery are reported in Figure 7. The balance in terms of N/P ratio of the full cell may be achieved from the comparison of half-cell voltage profiles in a steady-state cycle (i.e., fourth one) of the LFP cathode and Li_*y*_SiO_*x*_C anode reported in Figure 7a, galvanostatically tested at C/20 (LFP-MEA) and 0.1 mA cm^–2^ (Li_*y*_SiO_*x*_C) at 70 °C. It is worth mentioning that these profiles, taken from the cells discussed in the previous section, are compared taking into account the pure capacity (mAh) instead of the specific one (mAh g^–1^) to facilitate the N/P ratio determination. Therefore, the resulting chart shows a value of 0.380 mAh for Li-insertion into LFP (discharge in half-cell) and 0.441 mAh for Li-dealloying/deinsertion from Li_*y*_SiO_*x*_ (charge in half-cell), yielding a practical N/P ratio of 1.16 (see Experimental Section for further details). Since the SiO_*x*_C loading in the full-cell configuration (3.7 mg cm^–2^) is higher than that of the half-cell (3.2 mg cm^–2^), the prelithiation duration was adjusted accordingly by increasing the activation time from 2 to 2.5 h, to ensure a higher efficiency at the first cycle. The longer prelithiation time used herein compared to previous work is requested to form a suitable SEI at the anode/CP-EM interphase, which may subtract a notable amount of lithium reservoir, necessary instead for a suitable full-cell balance. The selected charge/discharge voltage profiles of the full cell at various C-rates are then presented in Figure 7b. The cell exhibits a sloping behavior, with charge extending from 3.05 to 3.75 V and discharge from 3.25 to 2.05 V, reflecting the combination of the multistep shape of the Li_*y*_SiO_*x*_C anode ascribed to Li (de)­alloying and (de)­insertion discussed above,^31^ and the flat one due to Li (de)­insertion into the LFP cathode. Figure 7b also reveals that the cell delivers initial charge and discharge capacities of 143 and 140 mAh g^–1^, respectively, corresponding to a high initial %CE of 98%, as also evidenced by the cycling trend of Figure 7c. This remarkable reversibility suggests again the effectiveness of the preactivation protocol of the anode, the MEA setup of the cathode, and the cell balancing strategy employed in this study. Furthermore, the successful operation of the full cell confirms that the CP-EM with high salt concentration can effectively operate in the potential window of 1.5–4.0 V, thereby enabling the practical coupling of the LFP cathode with the Li_*y*_SiO_*x*_C anode. However, after the first cycle at C/20, the %CE decreases to about 90% and the delivered capacity reaches ∼100 mAh g^–1^ upon the 5 charge/discharge runs, likely due to irreversible processes leading to the SEI consolidation already discussed in Figure 6. Further rise of the C-rate to C/10 leads to the expected polarization increase, efficiency fluctuation, and capacity decrease to ∼85 mAh g^–1^, while the capacity reaches ∼50 mAh g^–1^ at a higher current rate of C/5.

**Figure 7:**
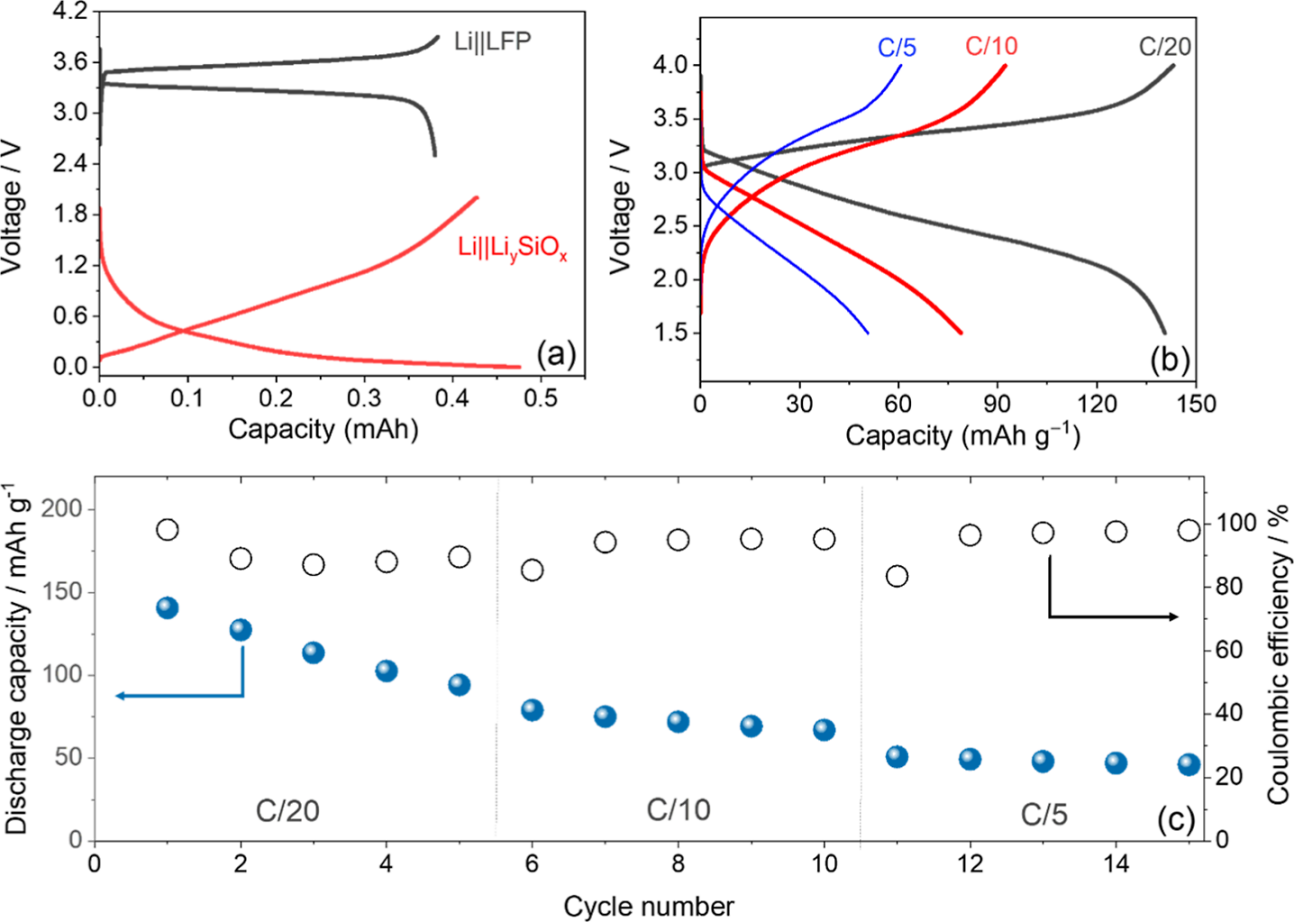
CP-EM in the Li_*y*_SiO_*x*_C|LFP-MEA Li-ion full cell. (a) Voltage profiles in Li half-cells in the 4th steady-state cycle, measured for comparison with 2.5 h activated Li_*y*_SiO_*x*_C at 0.1 mA cm^–2^ in the 0.01–2.0 V range, and LFP-MEA at C/20 (1C = 170 mA g^–1^) in the 2.5–3.9 V. (b) 1st charge/discharge voltage profiles and (c) cycling performance of the full-cell at increasing C-rates from C/20 to C/10 and C/5 in the 1.5–4.0 V range (%CE in right *y*-axis). LFP mass loading = 6 mg cm^–2^. Li_*y*_SiO_*x*_C mass loading = 3.7 mg cm^–2^. N/P capacity ratio = 1.16. Temperature: 70 °C.

Despite capacity decrease, efficiency fluctuation, and limited rate capability, the above data demonstrate for the first time the applicability of the proof-of-concept full cell in the solid polymer configuration at moderately high temperatures, such as the one adopted herein. On the other hand, the Li_*y*_SiO_*x*_C|LFP cell configuration can stand up to long cycling in conventional liquid electrolyte, as demonstrated by Figure S14 (Supporting Information) at C/5, while it is still limited by c-rate and stability in the polymer configuration.

## 4 Conclusion

This study successfully realized a robust, additive-free cross-linked polymer electrolyte membrane by synergistically blending poly­(ethylene carbonate/ethylene oxide) with LiFSI salt concentration increased up to 140 mol %. This pathway allowed a CP-EM with controlled molecular weight and cross-linkable unit density, as demonstrated by ^1^H NMR, and thermal stability approaching 170 °C that reproduced the TGA/DTG features of the pure LiFSI. In addition to thermal and mechanical stability, the membrane enabled low-polarization of the lithium stripping/plating for over 200 h at 70 °C at 0.1 mA cm^–2^. The ionic conductivity of the CP-EM ranged from 1.1 × 10^–4^ S cm^–1^ at 98 °C to 2.7 × 10^–7^ S cm^–1^, according to the VTF behavior, which allowed us to calculate *E*_a_ of 0.110 eV, significantly lower than that of Li^+^ motion in typical PEO-based electrolytes. Furthermore, the CP-EM exhibited a high *t* ^+^ of 0.61 and a wide electrochemical stability window extending from ∼0 V vs Li^+^/Li to above 4.0 V vs Li^+^/Li at 70 °C, suggesting its applicability in the 4 V-class Li-ion batteries. The suitability of the CP-EM has been initially demonstrated in a Li half-cell exploiting the MEA setup at the LFP cathode side and the prelithiated SiO_*x*_C composite at the anode side. The Li|CP-EM|LFP-MEA operated at temperatures ranging from 70 °C to values as high as 90 °C and delivered at C/10 reversible capacity ranging from 120 to 160 mAh g^–1^, however, with a rate-capability limited to C/5. On the other hand, the prelithiated Li_*y*_SiO_*x*_C achieved at 70 °C a reversible capacity approaching 300 mAh g^–1^, with fluctuating efficiency due to the consolidation of a SEI layer at the electrode/electrolyte interphase. Crucially, the practical features of the CP-EM at the solid state were demonstrated for the first time in an all-solid-state full-cell configuration operating at 70 °C, pairing the prelithiated silicon oxide-based anode with the LFP-MEA cathode. The cell exhibited charge from 3.05 to 3.75 V and discharge from 3.25 to 2.05 V, reflecting the combination of the multistep shape of the Li_*y*_SiO_*x*_C anode ascribed to Li (de)­alloying and (de)­insertion and the flat one due to Li (de)­insertion into the LFP cathode. Furthermore, the full cell delivered at C/20 an initial discharge capacity of 140 mAh g^–1^, with %CE as high as 98%, demonstrating the feasibility of the system without liquid plasticizers or any additives. However, the proof-of-concept full cell suffered from low rate capability and capacity decay upon cycling, thus suggesting the necessity of further improvements in ionic conductivity and electrochemical stability. To address these limitations, future research should focus on fine-tuning the macromolecular architecture and incorporating inorganic fillers into the membrane to increase the ionic conductivity. On the other hand, the relevant thermal stability and structural integrity of this cross-linked system suggest it as a suitable medium for practical applications in high-temperature environments, where safety and stability are prioritized over high room-temperature power density. Therefore, these findings may provide a significant breakthrough for the realization of safe and scalable solid-state lithium-ion batteries, based on cross-linked aliphatic poly­(ethylene carbonate/ethylene oxide) electrolytes.

## Supplementary Material



## Notes

Photographs illustrating the preparation process of CP-EM (Figure S1); schematic illustration of the fabrication process for symmetric/asymmetric cells with CP-EM (Figure S2); photographic image of LFP-MEA (Figure S3); cross-sectional SEM image of LFP-MEA (Figure S4); FT-IR spectra of the neat polymer, CP, and CP-EM (Figure S5); FT-IR spectra of the CP without salt and the CP-EM in the stretching vibration region of C–O–C and –SO_2_ (Figure S6); TGA curve of the CP-EM for N_2_ adsorption/desorption (Figure S7); DSC of the CP-EM (Figure S8); tensile stress–strain curves of the CP-EM (Figure S9) and related data summary (Table S1); Nyquist from EIS measurements of SS|CP-EM|SS symmetrical cell for ionic conductivity (Figure S10); analyses of Nyquist plots from EIS for ionic conductivity measurement (Table S2); VTF equation fit coefficients for the determination of the *E*_a_ (Table S3); Nyquist plots from EIS during aging of Li|CP-EM|Li symmetrical cells at 70 °C over 168 h (Figure S11) and related NLLS analyses (Table S4); CV of the Li|CP-EM|SPC-Cu cell and Li|CP-EM|Cu reference cell (Figure S12); CV/EIS study of the Li|CP-EM|LFP-MEA cell at 85 °C (Figure S13); and cycling response of a Li_*y*_SiO_*x*_C|LP30|LFP full-cell (Figure S14) (PDF)
