# Supplementary material for: Cross-Linked Poly(Ethylene Carbonate/Ethylene Oxide) Electrolyte for Solid-State Li-Ion Batteries Exploiting Membrane-Electrode Assembly
Source: ACS Polym Au. 2026 Jun 3;6(4):1072–87. doi: 10.1021/acspolymersau.6c00006 (PMC13474350; doi:10.1021/acspolymersau.6c00006)
Supplement: Supplementary file 1 [file lg6c00006_si_001.pdf]

Supporting Information

Cross-linked Poly(Ethylene Carbonate/Ethylene Oxide) Electrolyte for Solid-State Li-ion Battery Exploiting Membrane-Electrode Assembly

Nantapat Soontornnon[a], *Edoardo Barcaro*[d], *Shuto Takayama*[b], *Kento Kimura*[a,b], *Yoichi Tominaga*[a,c*], *and Jusef Hassoun*[a,d,e*]

[a]Institute of Global Innovation Research (GIR), Tokyo University of Agriculture and Technology, 3-8-1 Harumi-cho, Fuchu-shi, Tokyo 183-8538, Japan

[b]Department of Applied Chemistry, Graduate School of Engineering, Tokyo University of Agriculture and Technology, 2-24-16 Naka-cho, Koganei-shi, Tokyo 184-8588, Japan

[c]Graduate School of Advanced Interdisciplinary Science, Tokyo University of Agriculture and Technology, 2-24-16, Nakamachi, Koganei-shi, 184-8588, Tokyo

[d]Department of Chemical, Pharmaceutical and Agricultural Sciences University of Ferrara, Via Fossato di Mortara 17, 44121, Ferrara, Italy

[e]Center for Nanotechnology Innovation @NEST, Istituto Italiano di Tecnologia, Piazza San Silvestro 12, I-56127 Pisa, Italy

*Corresponding authors: jusef.hassoun@unife.it, ytominag@cc.tuat.ac.jp

Keywords

PEC, PEO, cross-linked polymer electrolyte, MEA, Li-ion battery

Contains 14 Figures and 4 Tables

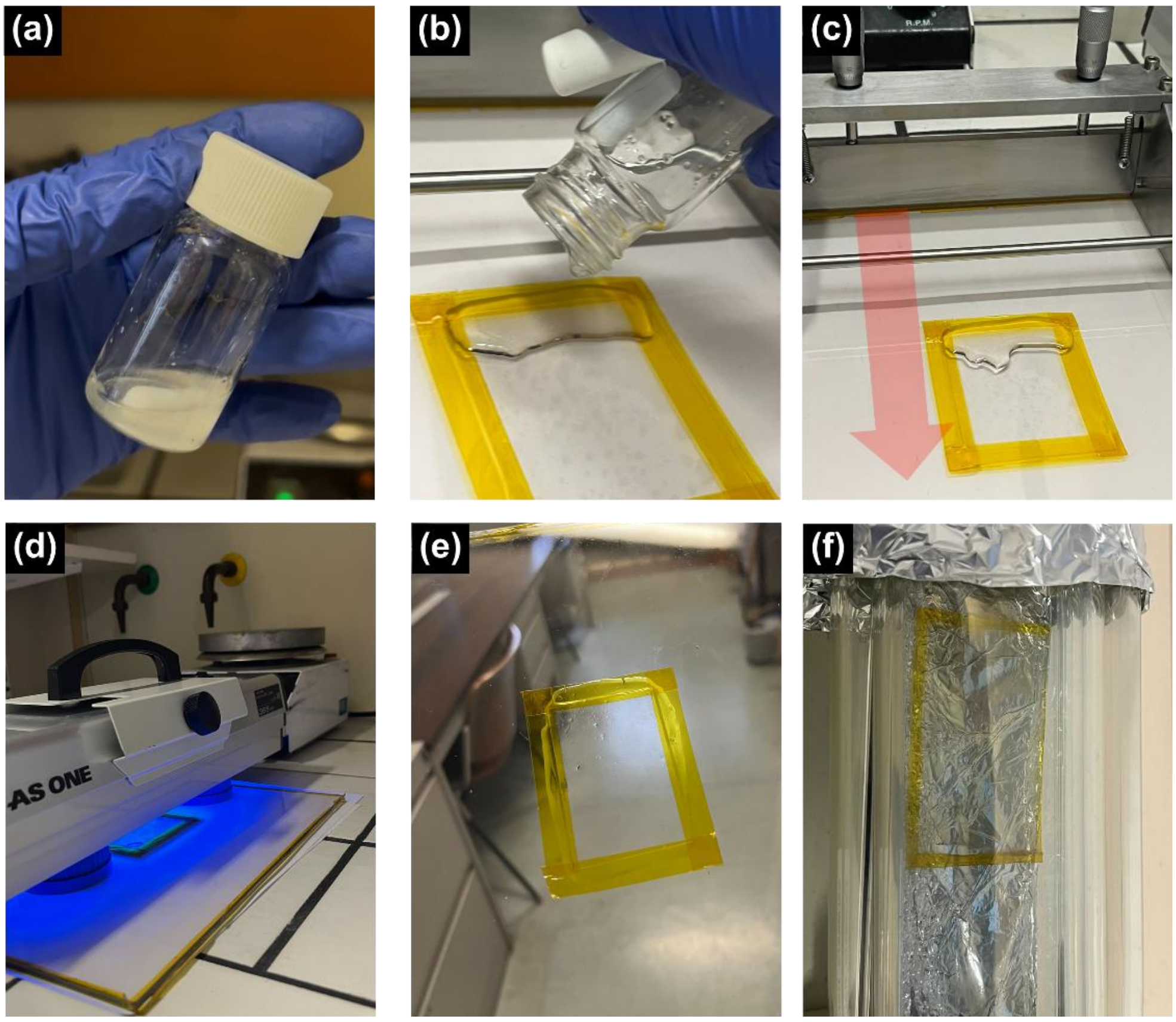

Figure S1. Photographs illustrating the preparation process of CP-EM: (a) electrolyte precursor, (b) pouring onto the FEP substrate, (c) film casting with a doctor blade (thickness: 600 μm), followed by (d) UV irradiation for 30 min to induce the crosslinking process, (e) formation of the gelled membrane, and (f) drying using a Büchi oven. See Manuscript text for acronyms.

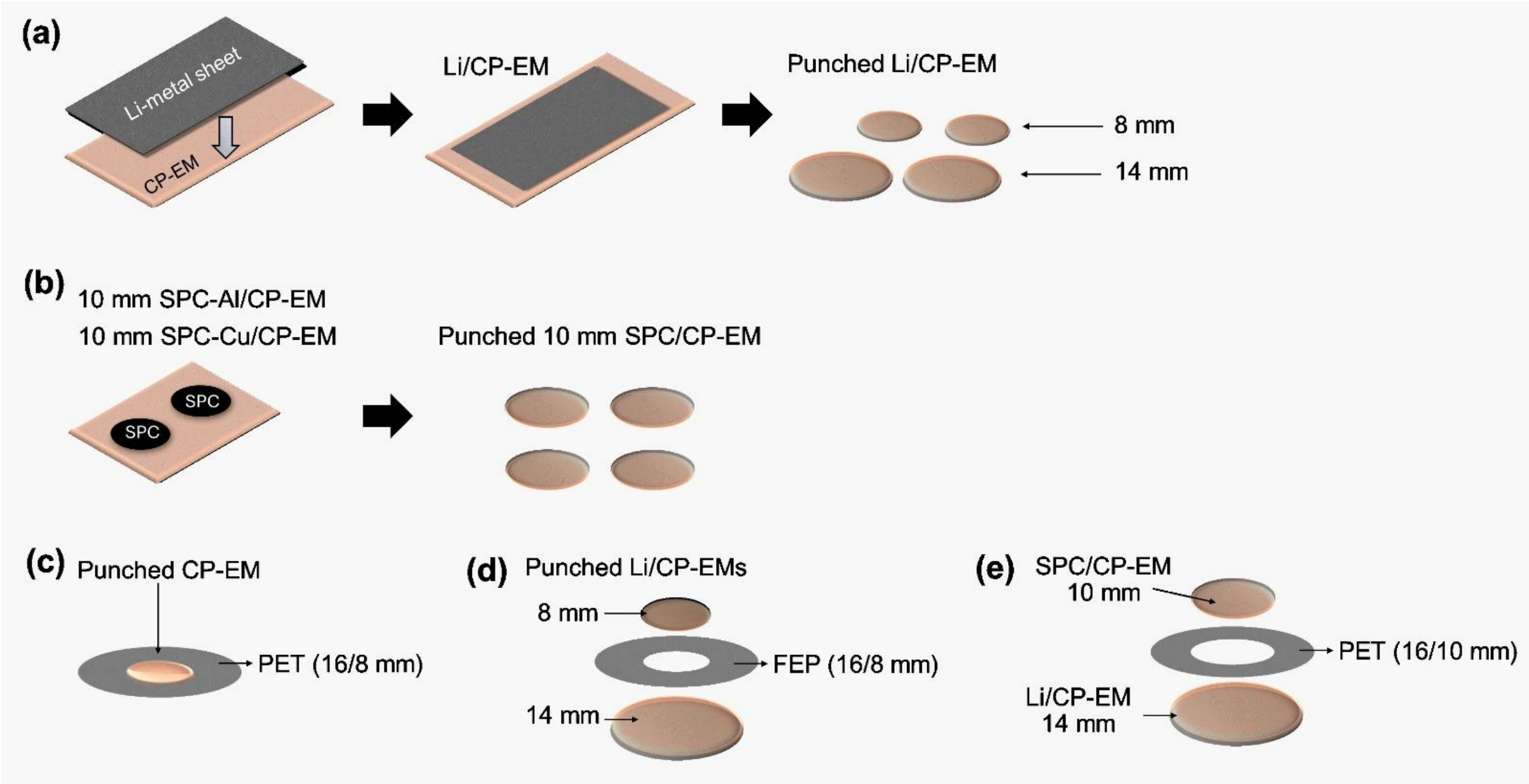

Figure S2. Schematic illustration of the fabrication process for SS|CP-EM|SS, Li|CP-EM|Li and Li|CP-EM|SPC cells. (a) Preparation of the laminated Li-metal sheet on CP-EM and cutting the CP-EM/Li laminate into disk-shaped electrodes before cell assembly. (b) Preparation/placing SPC-based electrodes on CP-EM and cutting into disks before cell assembly. (c) Ionic conductivity cell's preparation by fitting CP-EM into the inner diameter of PET O-ring. (d) Assembly of symmetric Li-cells using Li/CP-EM electrodes for interfacial stability and plating/stripping tests. (e) Assembly of cells using Li/CP-EM and SPC-based/CP-EM electrodes for anodic and cathodic stability tests. See Manuscript text for acronyms.

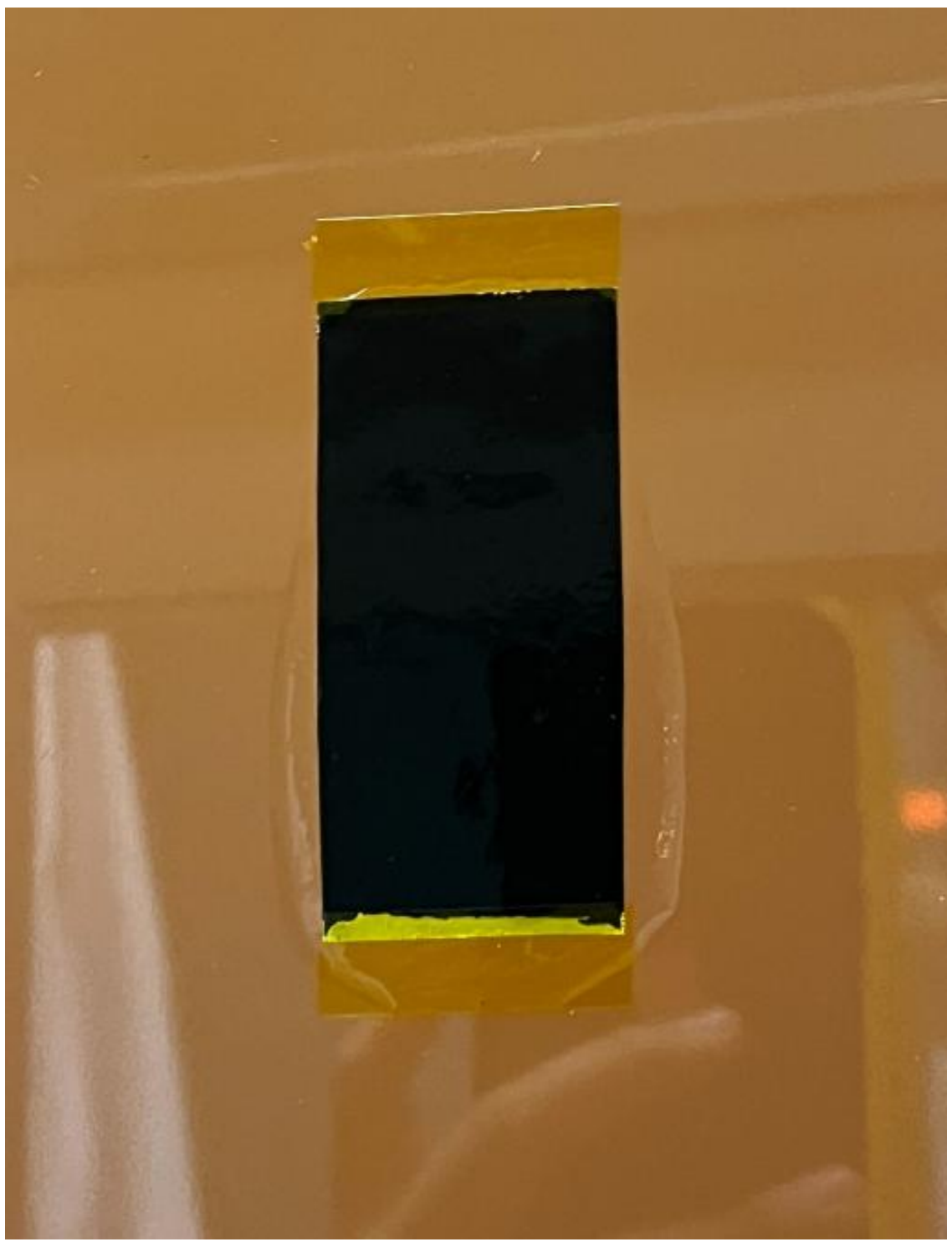

Figure S3. Photographic image of LFP-MEA (LFP mass loading = 6 mg cm^{-2}). See Manuscript text for acronyms.

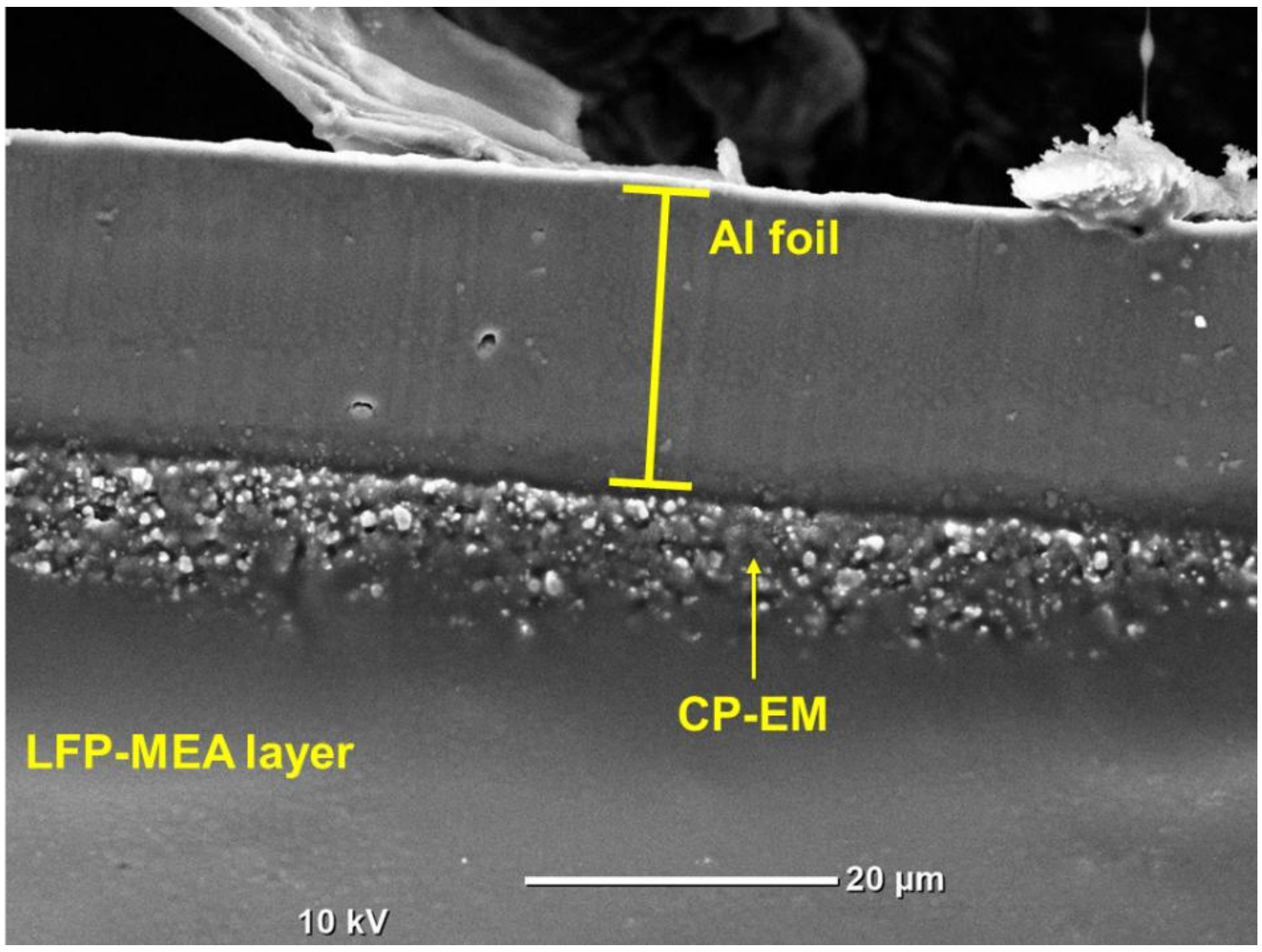

Figure S4. Cross-sectional SEM image of LFP-MEA. See Manuscript text for acronyms.

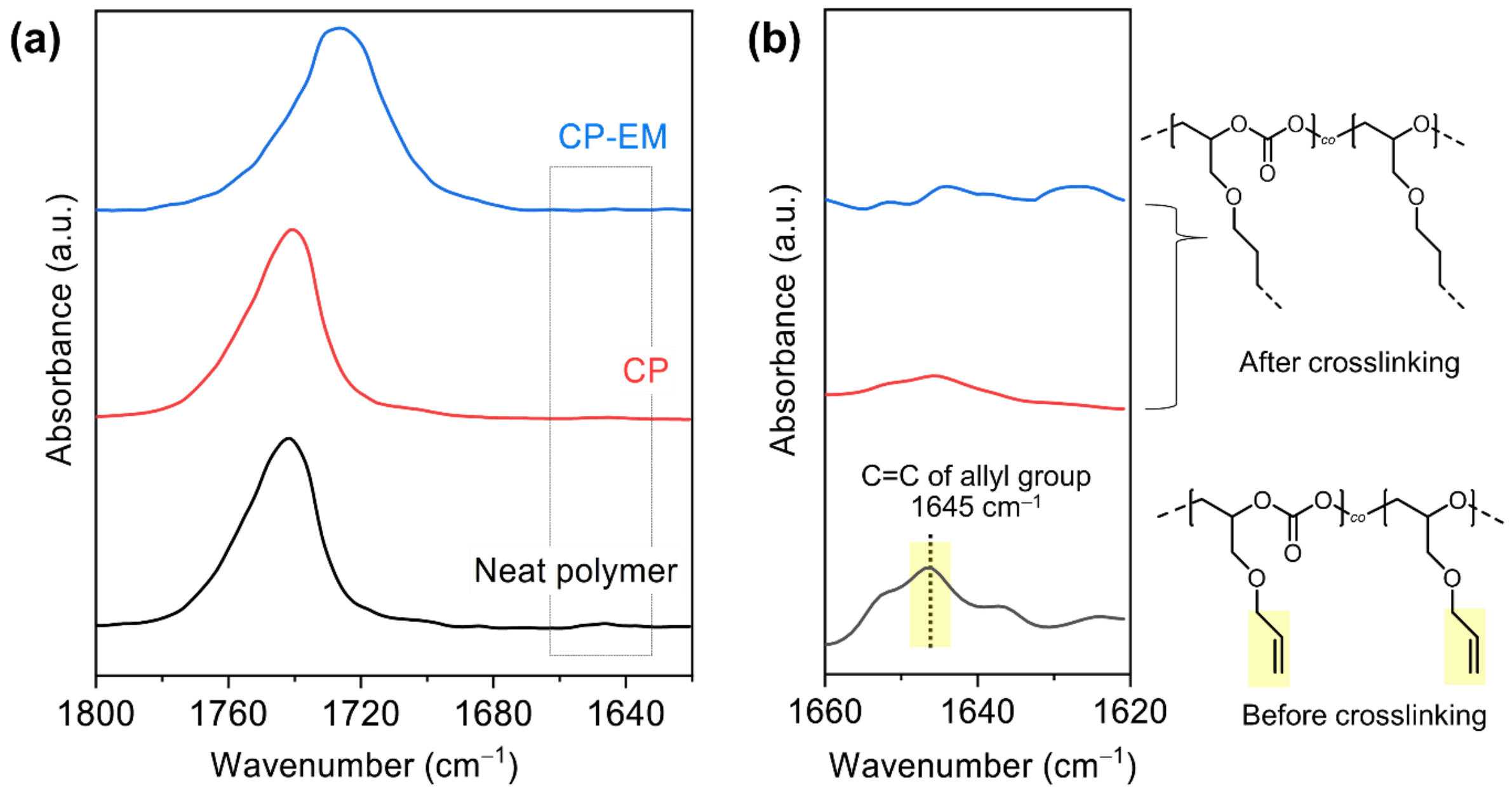

Figure S5. FT-IR spectra of the neat polymer (before crosslinking), CP (0 mol% LiFSI), and CP-EM (140 mol% LiFSI) in the stretching vibration region of **(a)** 1800 – 1620 cm^{-1} and **(b)** enlarged FT-IR spectra in the wavenumber range of 1660 – 1620 cm^{-1}, scheme of polymer before and after crosslinking. See Manuscript text for acronyms.

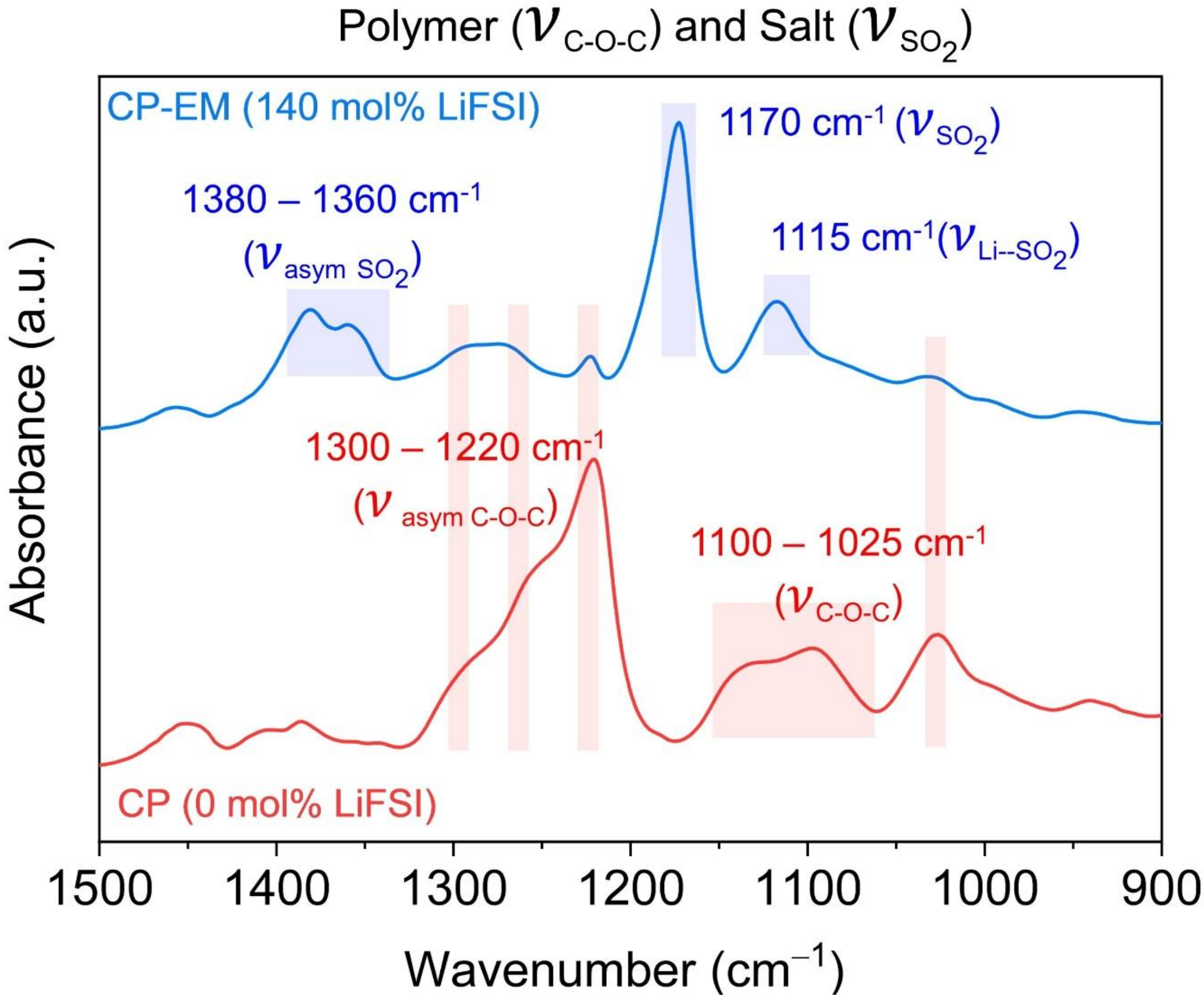

Figure S6. FT-IR spectra of CP (0 mol% LiFSI, red) and CP-EM (140 mol% LiFSI, blue) collected in the wavenumber range 1500−900 cm^{-1}, indicating the stretching vibration modes of the C-O-C ($\upsilon_{C\text{-}O\text{-}C}$) of the copolymer and SO_2 (υ_{SO2}) of the FSI anion. See Manuscript text for acronyms.

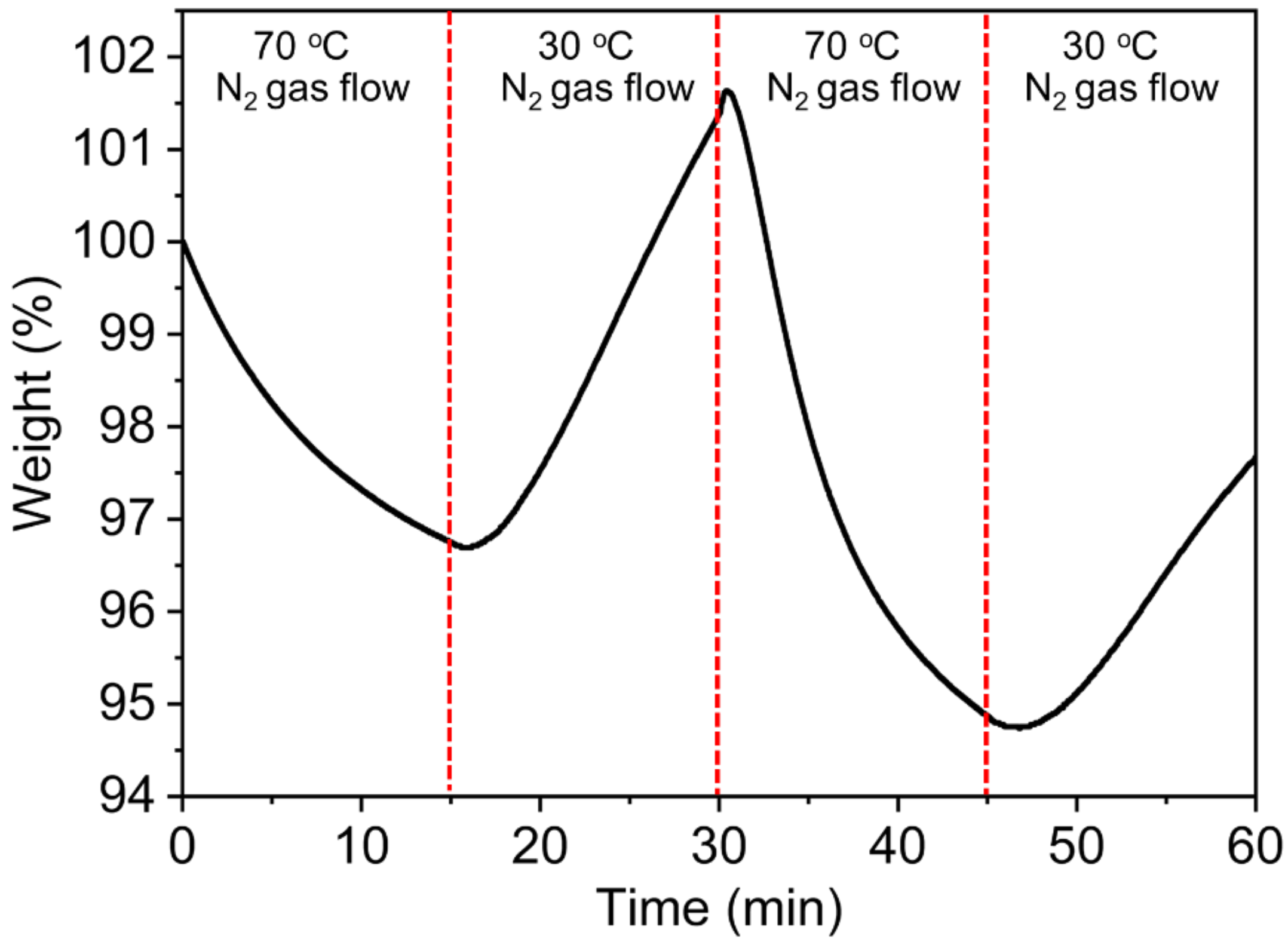

Figure S7. TGA curve of CP-EM showing weight (%) versus time (min) under N_2 flow (50 mL min^{-1}) with isotherms held at 30 °C and 70 °C. See Manuscript text for acronyms.

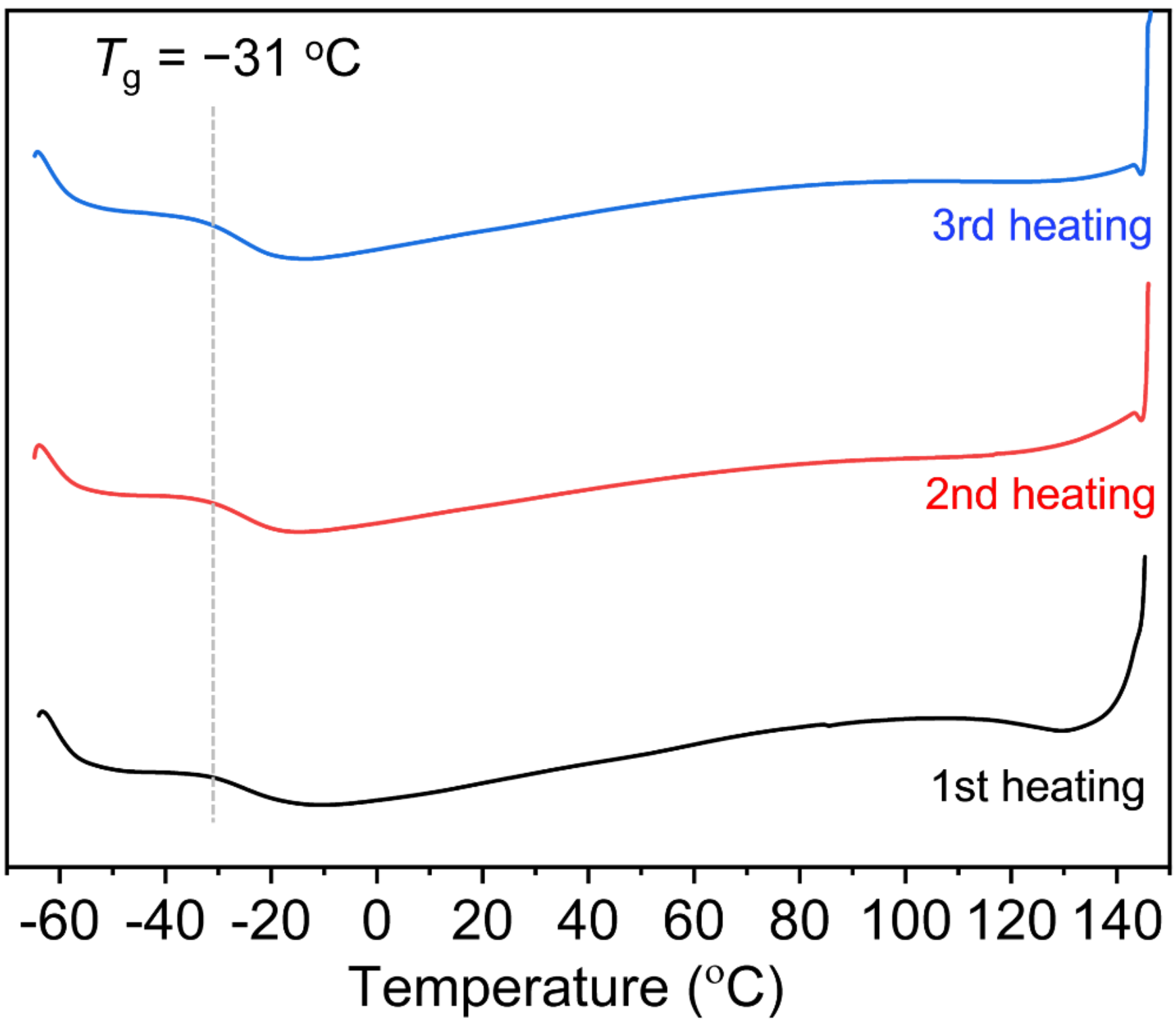

Figure S8. DSC thermograms of the CP-EM. Measurements conducted under N_2 flow of 40 mL min^{-1} for 3 cycles in temperature range from −65 to 145 °C, with heating/cooling rate of 10 °C/min. See Manuscript text for acronyms.

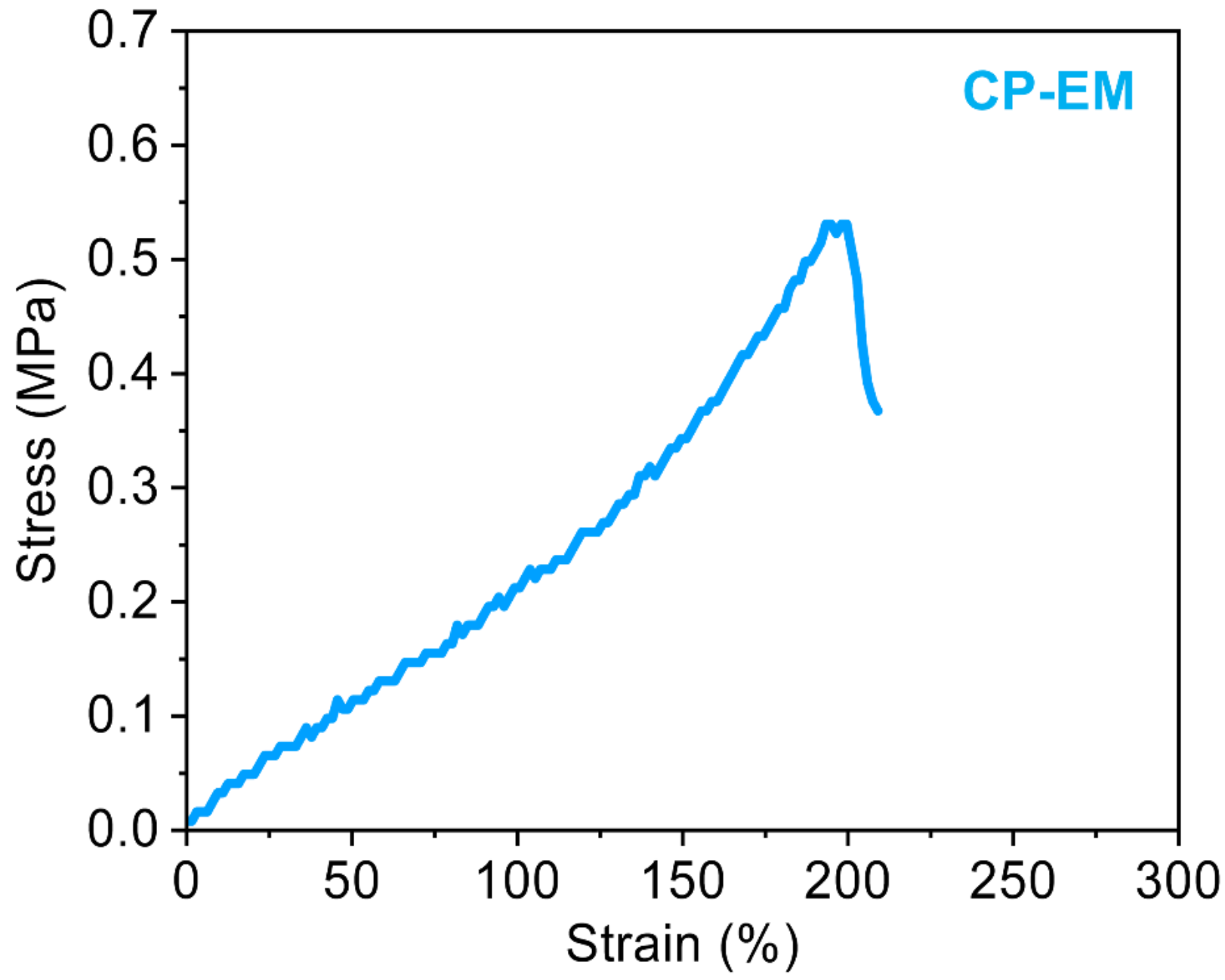

Figure S9. Representative result of stress–strain curves of CP-EM measured by tensile test using a crosshead speed of 10 mm min^{-1} at room temperature. See Manuscript text for acronyms.

Table S1. Evaluated mechanical properties from tensile stress-strain curve in Figure S9

Sample	Maximum stress (MPa)	[a]Young's modulus (MPa)	Elongation at break (%)
CP-EM	0.53±0.19	0.20±0.08	210±20

[a] estimated using the initial slope of the tensile stress-strain curve, with mean ± standard deviation (n = 5).

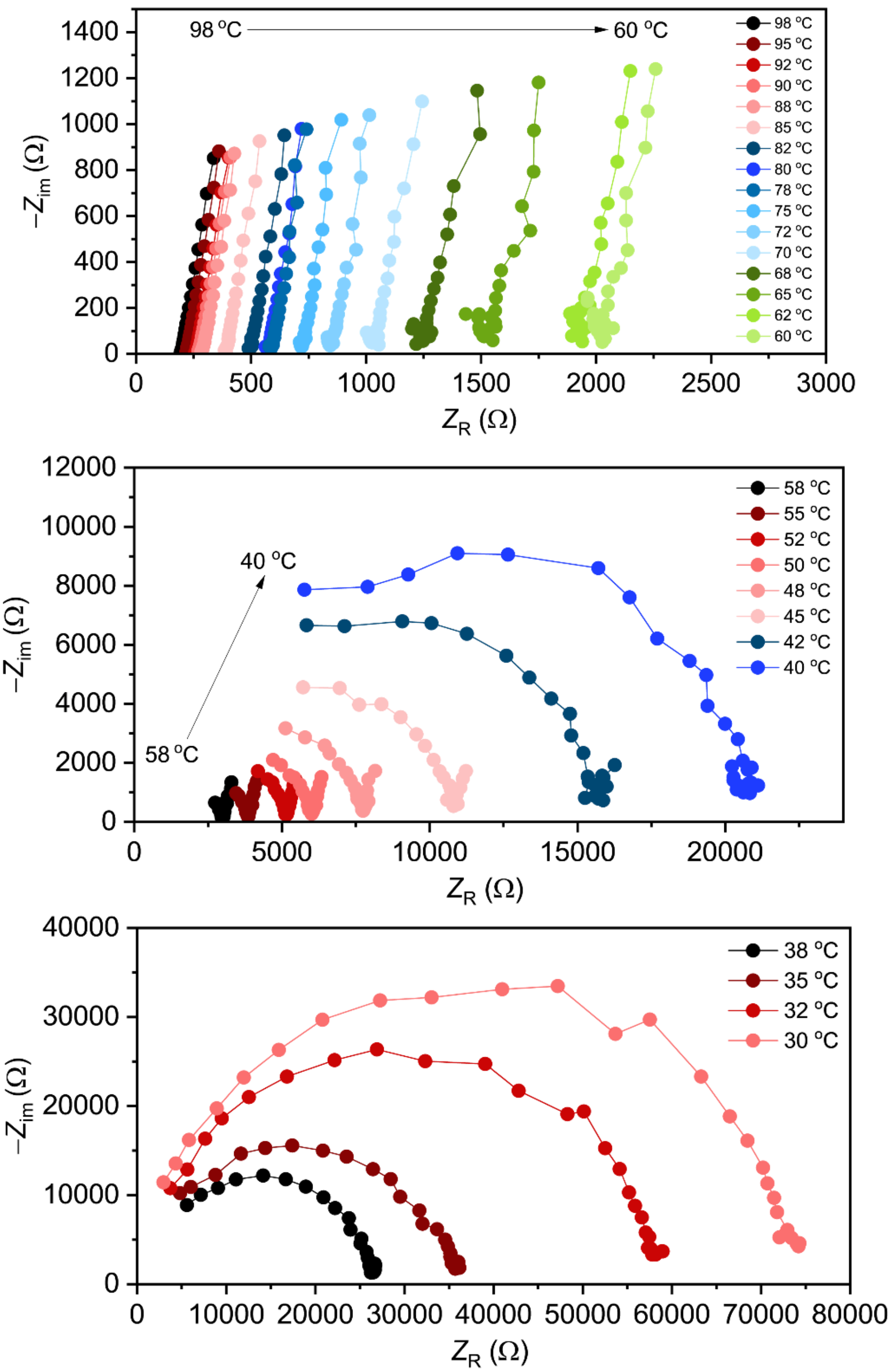

Figure S10. Nyquist plots obtained from electrochemical impedance spectroscopy (EIS) recorded over a temperature range from 98 °C to 30 °C with a decrement of 2–3 °C, using a SS|CP-EM|SS symmetrical cell to assess the ionic conductivity of the electrolyte (Figure 4a in Manuscript). Detailed experimental procedures and data analysis methods are provided in the Experimental Section of the main Manuscript.

Table S2. NLLS Analyses performed on the Nyquist plots of Figures S10, recorded by EIS on the SS|CP-EM|SS blocking electrode cell used for determining the ionic conductivity (***σ***) in temperature range from 98 °C to 30 °C. Ionic conductivity calculated using equation (1) in the Manuscript from fitted values. The ionic conductivity is reported against the temperature in Figure 4a of Manuscript. Only χ^2 values of the order of 10^{-4} or below are considered suitable for analysis.

**Temperature** (°C)	**Fitting circuit**	R_b **(Ω)**	σ **S cm^{-1}**	χ^2
98	$R_{b1}Q_g$	198	1.01×10^{-4}	4.1×10^{-5}
95	$R_{b1}Q_g$	220	9.09×10^{-5}	7.7×10^{-5}
92	$R_{b1}Q_g$	252	7.94×10^{-5}	5.6×10^{-5}
90	$R_{b1}Q_g$	269	7.43×10^{-5}	7.5×10^{-5}
88	$R_{b1}Q_g$	282	7.09×10^{-5}	1.9×10^{-5}
85	$R_{b1}Q_g$	376	5.32×10^{-5}	2.3×10^{-4}
82	$R_{b1}Q_g$	465	4.30×10^{-5}	2.2×10^{-4}
80	$R_{b1}Q_g$	488	4.10×10^{-5}	2.7×10^{-4}
78	$R_{b1}Q_g$	562	3.56×10^{-5}	4.7×10^{-4}
75	$R_{b1}Q_g$	711	2.81×10^{-5}	5.2×10^{-4}
72	$R_{b1}Q_g$	810	2.47×10^{-5}	1.5×10^{-4}
70	$R_{b1}(R_{b2}Q_{b2})Q_g$	877	2.28×10^{-5}	6.6×10^{-4}
68	$R_{b1}(R_{b2}Q_{b2})Q_g$	1247	1.60×10^{-5}	1.3×10^{-4}
65	$R_{b1}(R_{b2}Q_{b2})Q_g$	1500	1.33×10^{-5}	1.7×10^{-4}
62	$R_{b1}(R_{b2}Q_{b2})Q_g$	1900	1.05×10^{-5}	3.6×10^{-4}
60	$R_{b1}(R_{b2}Q_{b2})Q_g$	2450	8.16×10^{-6}	7.1×10^{-5}
58	$R_{b1}(R_{b2}Q_{b2})Q_g$	2888	6.93×10^{-6}	4.8×10^{-5}
55	$R_{b1}(R_{b2}Q_{b2})Q_g$	3851	5.19×10^{-6}	9.1×10^{-5}
52	$R_{b1}(R_{b2}Q_{b2})Q_g$	5146	3.89×10^{-6}	7.8×10^{-5}
50	$R_{b1}(R_{b2}Q_{b2})Q_g$	6046	3.31×10^{-6}	9.4×10^{-5}
48	$R_{b1}(R_{b2}Q_{b2})Q_g$	7732	2.59×10^{-6}	1.8×10^{-4}
45	$R_{b1}(R_{b2}Q_{b2})Q_g$	10763	1.86×10^{-6}	1.5×10^{-4}
42	$R_{b1}(R_{b2}Q_{b2})Q_g$	15591	1.28×10^{-6}	3.9×10^{-4}
40	$R_{b1}(R_{b2}Q_{b2})Q_g$	20836	9.60×10^{-7}	9.8×10^{-4}
38	$R_{b1}(R_{b2}Q_{b2})Q_g$	26520	7.54×10^{-7}	1.9×10^{-4}
35	$R_{b1}(R_{b2}Q_{b2})Q_g$	36152	5.53×10^{-7}	6.3×10^{-4}
32	$R_{b1}(R_{b2}Q_{b2})Q_g$	58129	3.44×10^{-7}	6.3×10^{-4}
30	$R_{b1}(R_{b2}Q_{b2})Q_g$	74208	2.70×10^{-7}	6.8×10^{-4}

Table S3. VTF equation fit coefficients (eq. 3 in Manuscript) for the determination of the E_a from the conductivity plots of Figure 4a in the main Manuscript.

**Model**	**VTF Model**
Equation	y=a×exp(b×x/(c−x))
a	0.432 ± 0.16
B	−6.279 ± 0.50
c	4.741 ± 0.07
Reduced Chi-Sqr	0.328
R-Square (COD)	0.996

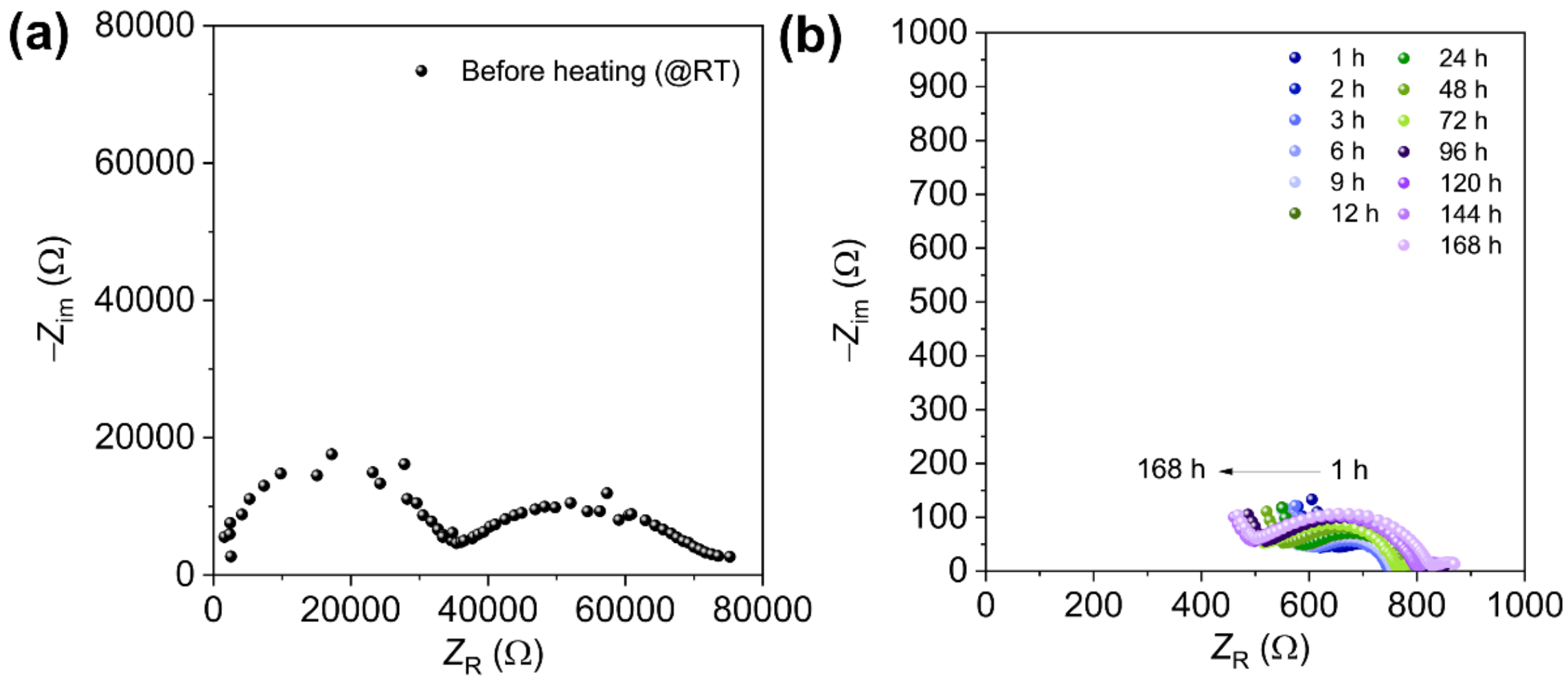

Figure S11. Nyquist plots obtained from electrochemical impedance spectroscopy (EIS) periodically recorded during the aging of Li|CP-EM|Li symmetrical cells over 168 h after assembly. Measurements conducted **(a)** at room temperature prior to aging at 70 °C and **(b)** at 70 °C. The EIS spectra were collected over a frequency range of 500 kHz to 100 mHz with an applied AC amplitude of 10 mV. The evolution of interfacial resistance is presented in Figure 4c of the main Manuscript, and detailed procedures for cell preparation and measurement are described in the Experimental Section.

Table S4. Non-linear least-squares (NLLS) analyses performed on the Nyquist plots of Figures S11 recorded by EIS in Li|CP-EM|Li symmetrical cell under aging at 70 ºC for 168 h (1 week). The fitted values are reported in the resistance trend displayed in Figure 4c in the Manuscript.

**Cell conditions**	**Fitting circuit**	$\boldsymbol{R_b}$ **(Ω)**	$\boldsymbol{R_i}$ **(Ω)** $\sum \boldsymbol{R_n}$	χ^2
After assembly (at RT)	$R_b(R_2Q_2)(R_3Q_3)(R_4Q_w)$	32,300	41,420	9.9×10^{-4}
1 h	$R_b(R_2Q_2)(R_3Q_3)(R_4Q_w)$	636	165	9.6×10^{-6}
2 h	$R_b(R_2Q_2)(R_3Q_3)(R_4Q_w)$	621	173	1.2×10^{-5}
3 h	$R_b(R_2Q_2)(R_3Q_3)(R_4Q_w)$	603	179	2.7×10^{-6}
6 h	$R_b(R_2Q_2)(R_3Q_3)(R_4Q_w)$	585	210	1.0×10^{-6}
9 h	$R_b(R_2Q_2)(R_3Q_3)(R_4Q_w)$	575	221	1.2×10^{-5}
12 h	$R_b(R_2Q_2)(R_3Q_3)(R_4Q_w)$	571	238	3.9×10^{-6}
24 h	$R_b(R_2Q_2)(R_3Q_3)(R_4Q_w)$	565	256	2.4×10^{-6}
48 h	$R_b(R_2Q_2)(R_3Q_3)(R_4Q_w)$	533	295	4.4×10^{-6}
72 h	$R_b(R_2Q_2)(R_3Q_3)(R_4Q_w)$	505	315	5.0×10^{-6}
96 h	$R_b(R_2Q_2)(R_3Q_3)(R_4Q_w)$	507	356	4.8×10^{-6}
120 h	$R_b(R_2Q_2)(R_3Q_3)(R_4Q_w)$	487	372	3.8×10^{-6}
144 h	$R_b(R_2Q_2)(R_3Q_3)(R_4Q_w)$	480	383	4.2×10^{-6}
168 h	$R_b(R_2Q_2)(R_3Q_3)(R_4Q_w)$	490	396	4.6×10^{-6}

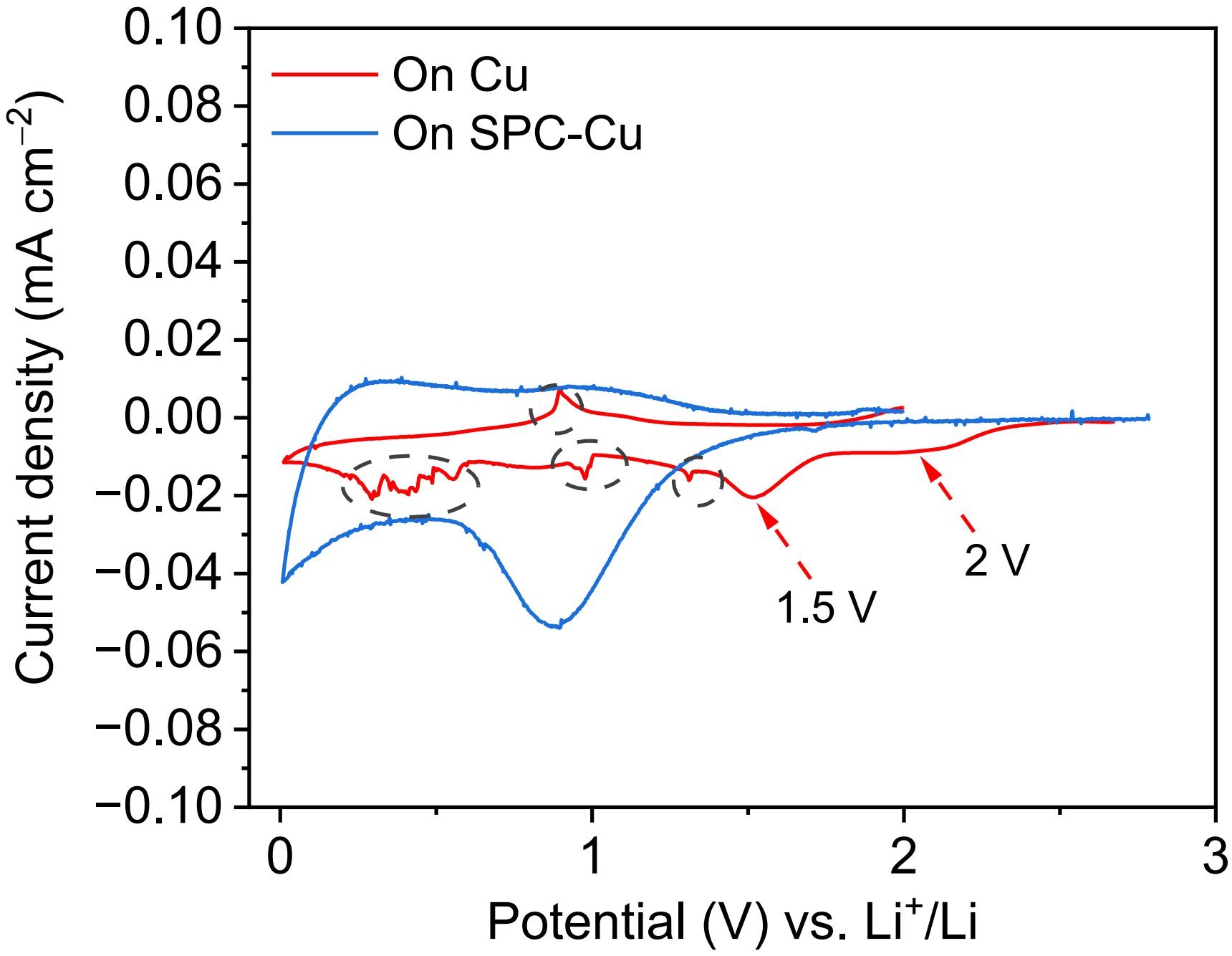

Figure S12. CV in the 0.01–2.0 V vs. Li^+/Li range using Li|CP-EM|SPC-Cu cell (blue, from Figure 4d in Manuscript) and a reference Li|CP-EM|Cu cell (red) at 70 ºC at a scan rate of 0.1 mV s^{-1}. See Manuscript text for acronyms.

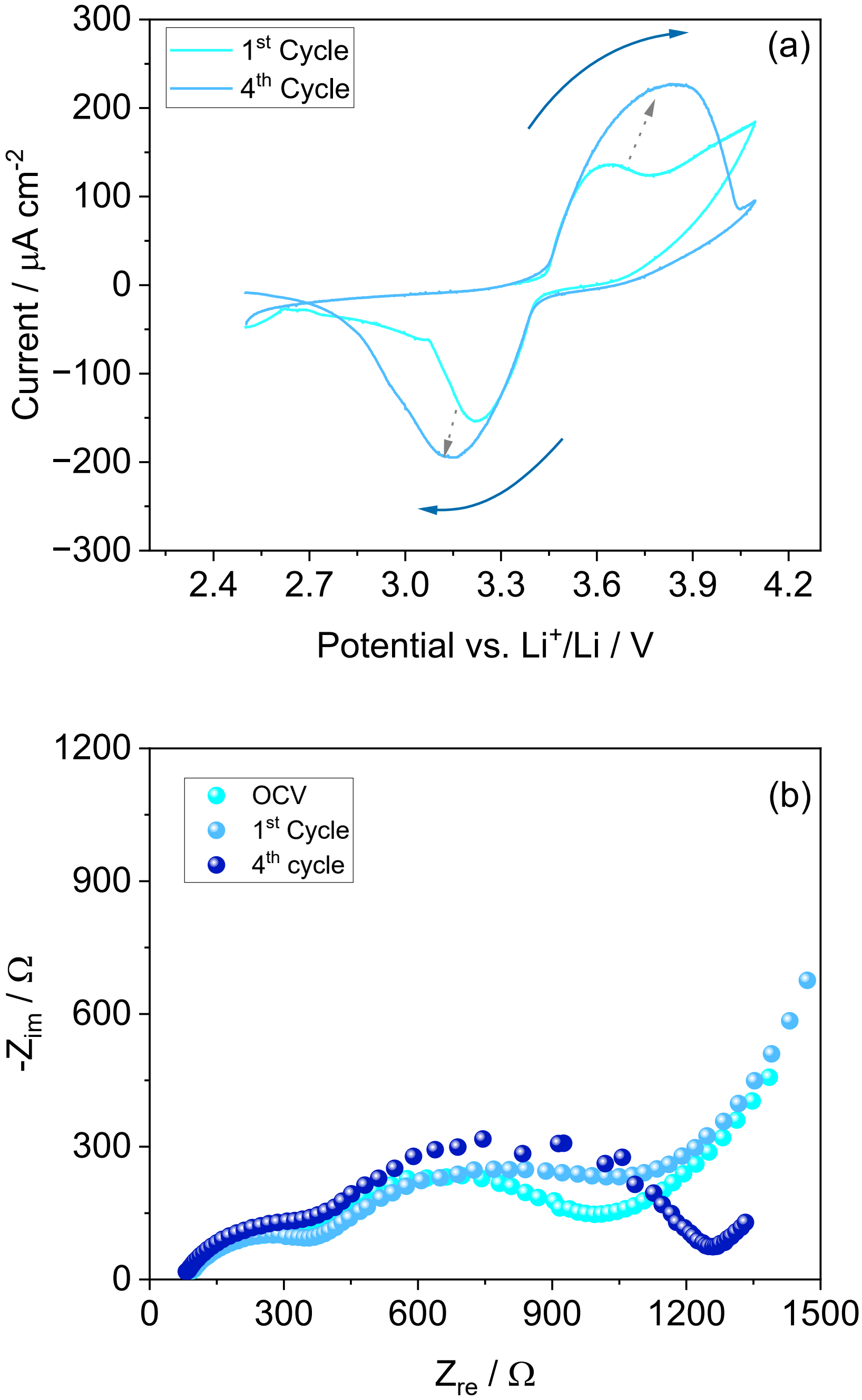

Figure S13. CV in the 2.5–4.1 V vs. Li^+/Li range of the Li|CP-EM|LFP-MEA cell at 85 °C, and related EIS at the OCV of the cell, after 1 and 4 cycles. Scan rate 0.1 mV S^{-1}. EIS with AC amplitude of 10 mV, in the frequency range of 500 kHz–100 mHz. See Manuscript text for acronyms.

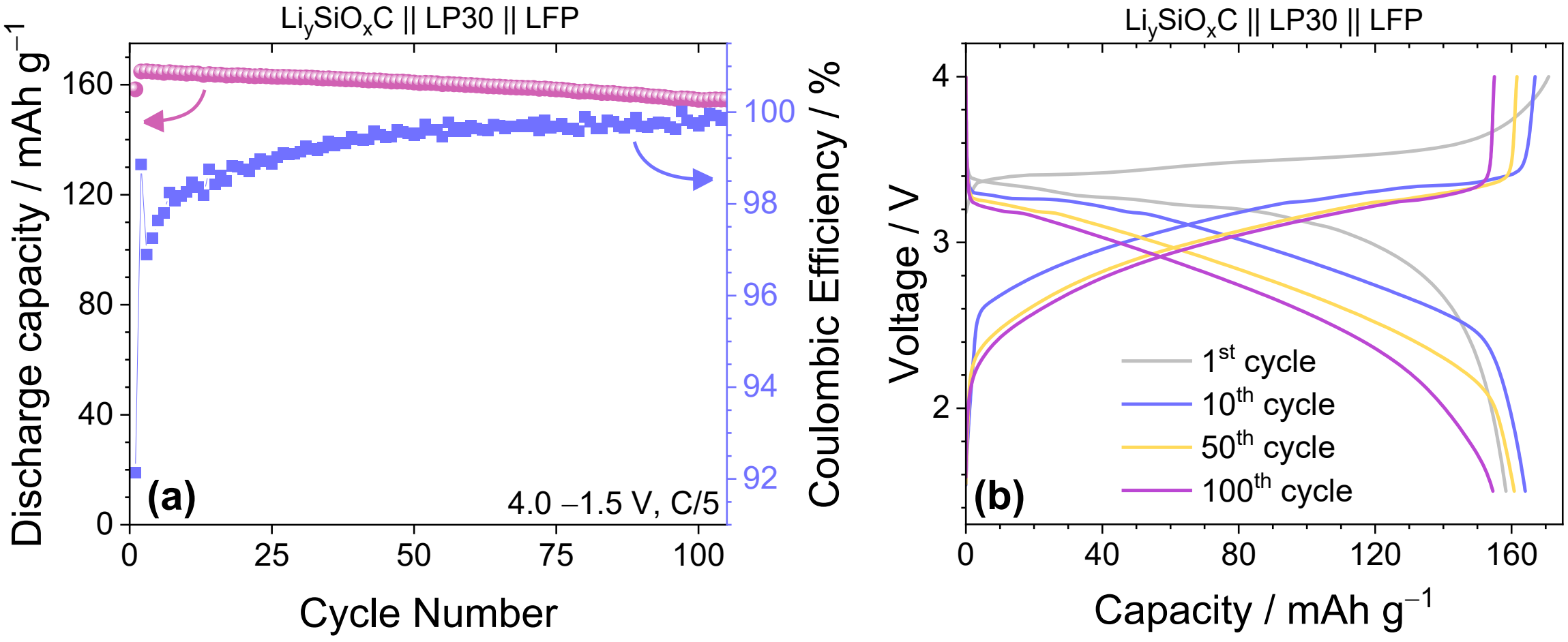

Fig S14. Galvanostatic cycling performance of Li_ySiO_xC|LP30|LFP cell at constant current of 0.2 C in terms of **(a)** discharge capacity trends versus cycle number (Coulombic efficiency represented on right *y*-axis of panel), **(b)** selected voltage profiles, active material mass loading 6.1 mg cm^{-2} and 3.3 mg_{SiOxC} cm^{-2} (electrode geometric area 0.785 cm^2 and 1.54 cm^2). The anode pre-lithiation process was applied for 20 min. Room temperature (25°C). See Experimental Section for acronyms.
